# Sulfide Quinone Oxidoreductase Alleviates Acute Ulcerative Colitis by Regulating Mitochondrial Dysfunction

**DOI:** 10.1002/mco2.70285

**Published:** 2025-07-13

**Authors:** Hailin Ma, Shuilian Fu, Chujun Huang, Na Han, Fangfang Cai, Dangran Li, Jian Cheng, Hongqin Zhuang, Zi‐Chun Hua

**Affiliations:** ^1^ The State Key Laboratory of Pharmaceutical Biotechnology, College of Life Sciences Nanjing University Nanjing PR China; ^2^ Jiangsu Key Laboratory of Neuropsychiatric Diseases & Institute of Neuroscience Soochow University Suzhou PR China; ^3^ Changzhou High‐Tech Research Institute of Nanjing University and Jiangsu Target Pharma Laboratories Inc. Changzhou PR China; ^4^ Faculty of Pharmaceutical Sciences Xinxiang Medical University Xinxiang PR China

**Keywords:** intestinal epithelial cells, mitochondrial dynamics, ROS, sulfide quinone oxidoreductase, ulcerative colitis

## Abstract

Alteration in mitochondrial function within intestinal epithelial cells were closely related to inflammatory bowel disease (IBD) progression. Sulfide quinone oxidoreductase (*SQOR*), located in the inner mitochondria membrane, is a crucial enzyme in sulfide metabolism. Here, we observed that *SQOR* was downregulated during colitis. Intestinal epithelial cells specific knockout of *SQOR* (*Sqor* ^CKO^) mice were more susceptible to acute ulcerative colitis (UC) with lower hydrogen sulfide (H_2_S) levels, and the absence of *SQOR* caused a breakdown of the epithelial barrier through disruption of the tight junction proteins. Furthermore, analysis of the mitochondrial morphology and functions revealed increased mitochondrial damage when *SQOR* deficiency. Mechanistically, it is observed that *SQOR* knockout increased lipid peroxidation, malondialdehyde (MDA) levels and ferroptosis. Further results demonstrated that *SQOR* may rely on inhibiting excessive mitochondrial division and promoting mitochondrial biogenesis to regulate reaction oxygen species (ROS) levels in intestinal epithelial cells. Treatment with ROS scavengers (NAC) showed significant reduced colonic inflammation symptoms observed in DSS‐treated *Sqor* ^CKO^ mice. Collectively, these findings demonstrate the protective role of *SQOR* in intestinal epithelial cells in maintaining mitochondrial homeostasis by regulating ROS and providing novel insight into UC.

## Graphical abstract

*SQOR* deficiency in intestinal epithelial cells aggravated DSS‐induced acute colitis and disrupted the integrity of intestinal tight junctions. Moreover, *SQOR* plays a vital role in regulating the mitochondrial function of intestinal epithelial cells. Mechanistically, *SQOR* may rely on inhibiting excessive mitochondrial division and promoting mitochondrial biogenesis to regulate ROS levels in intestinal epithelial cells.

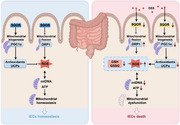

## 1 Introduction

Inflammatory bowel disease (IBD) is a chronic relapsing gastrointestinal tract inflammation and comprises two main forms ulcerative colitis (UC) and Crohn's disease [1]. IBD incidence has started to rise globally, and current treatment maintains remission in a small percentage of individuals, failing to control inflammation fully and IBD relapse [2]. UC is an idiopathic, superficial mucosal inflammation of the colorectum that starts from the rectum and rapidly continuously spreads to the proximal through part of or the entire colon, which is more prevalent than Crohn's disease [3, 4]. Specifically, UC is a multifactorial disease with an underlying cause associated with a disorder of epithelial barrier functions, immune response, genetic and environmental factors [5]. In clinical terms, the main features of UC are abdominal pain, rectal bleeding, and diarrhea [6]. However, the etiological mechanisms of UC remain incompletely characterized, but growing evidence shows the importance of the intestinal mucus barrier in disease pathogenesis [7].

Due to the high energy dependency of intestinal epithelial cells, mitochondrial dysfunction can impair mucosal integrity and intestinal stem cells self‐renewal, ultimately leading to epithelial cell apoptosis and exacerbating conditions such as IBD [8]. IBD has been related to disruptions in mitochondrial function. Specifically, genome‐wide association analyses identified *Slc25a28* and *Park7* as IBD risk factors and susceptibility genes encoding mitochondrial proteins [9]. Recently, a study reported markedly suppressing mitochondrial genes and function across cohorts of 206 UC patients [10, 11]. As such, mitochondrial dysfunction has been established as a critical factor in IBD pathogenesis, although their underlying mechanisms remain poorly understood [9].

Ferroptosis is regulated necrosis triggered by iron accumulation excessive lipid peroxidation and subsequent accumulation of reactive oxygen species (ROS) [6]. Lipid peroxide accumulation can occur through various mechanisms, including intracellular iron overload leading to ROS production, and loss of glutathione (GSH) peroxidase 4 (GPX 4) activity [12]. Emerging evidence demonstrated that ferroptosis in intestinal disease contributes to UC development and relieves colitis symptoms [13]. Studies have revealed that increased iron consumption is related to the pathogenesis of UC, and high iron intake is related to UC development in newly diagnosed patients [12].

Sulfide quinone oxidoreductase (*SQOR*) is a protein of the mitochondria membrane and is a crucial enzyme in sulfide metabolism [14]. *SQOR* catalyzed the first step in hydrogen sulfide (H_2_S) oxidation, converting sulfide to persulfate [15]. H_2_S represents an inorganic compound capable of driving mitochondrial ATP synthesis [16]. Among all cell types, intestinal epithelial cells demonstrate the highest efficiency in utilizing H₂S for bioenergetic functions [17]. Moreover, some studies have reported *SQOR* expression decreased in the UC patient rectum compared to healthy controls [16]. Although current data remain limited, preliminary studies indicated a potential association between *SQOR* and UC development [16, 18]. Furthermore, studies demonstrated that persulfate generated via *SQOR*‐mediated sulfide oxidation may act as an electron acceptor for the electron transfer chain (ETC) and promote mitochondrial adenosine triphosphate (ATP) production [19]. Recently, it has been observed that *SQOR*, the mitochondrial enzyme catalyzing hydrogen sulfide to reactive sulfur species (RSS), is essential for maintaining mitochondria homeostasis [20]. Although some studies have reported that *SQOR* was associated with neurological disorders and acute kidney injury (AKI) and where it alleviated AKI via influencing mitochondrial dysfunction [14], its exact function in the pathogenesis of colitis remains unclear.

In this study, we reported that *SQOR* was downregulated in colonic tissues of dextran sodium sulfate (DSS)‐induced acute UC model mice. We further showed that *Sqor* ^CKO^ mice aggravated DSS‐induced acute colitis and disrupted of intestinal tight junction integrity. Mechanistically, *SQOR* critically regulates the mitochondrial function in intestinal epithelial cells. Mainly, *SQOR* may rely on inhibiting excessive mitochondrial division and promoting mitochondrial biogenesis to regulate ROS levels in intestinal epithelial cells. Thus, *SQOR* may be a therapeutic target for UC associated with mitochondrial dysfunction.

## 2 Results

### 2.1 Downregulation of *SQOR* Is Accompanied by Acute UC Disease Progression in Mice

**FIGURE 1:**
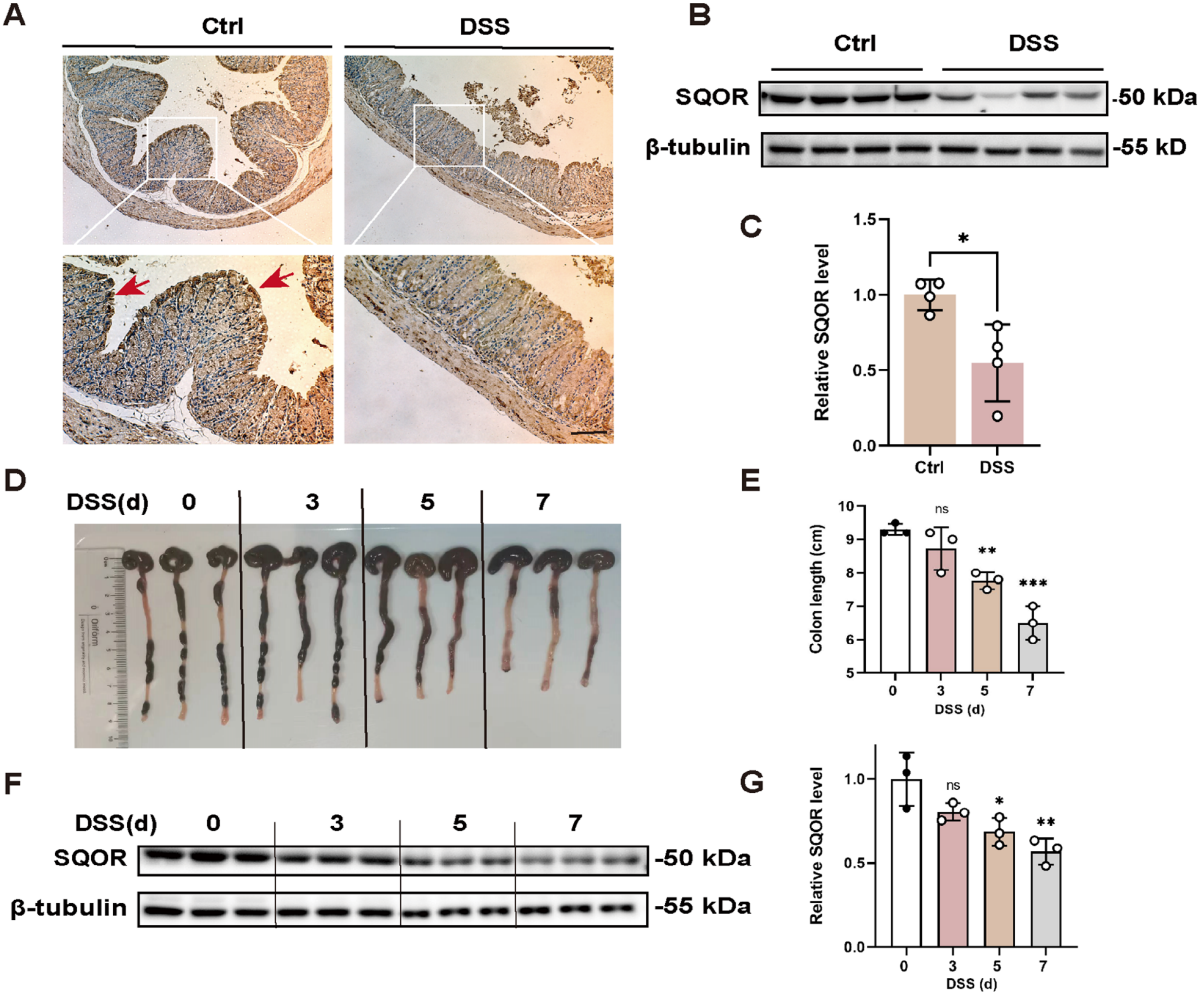
DSS‐induced acute UC significantly reduced SQOR levels. (A) C57BL/6 mice were treated with 3% DSS or water for 7 days, representative immunohistochemical (IHC) staining of *SQOR* in the colon, red arrows indicated intestinal epithelial cells (scale bar: 200 µm; enlarged scale bar: 100 µm; *n* = 3). (B, C) Mice were treated with 3% DSS for 7 days, and then colon was prepared to detect *SQOR* expression using western blot (*n* = 4). (D, E) Mice were treated with 3% DSS for 3, 5, and 7 days, representative photograph of colon tissue during DSS‐induced acute UC in colon tissue of WT mice, and the colon length was recorded at days 0, 3, 5, 7 (*n* = 3). (F, G) Relative *SQOR* protein expression in colonic tissues from controls or DSS‐treated WT mice during days 0, 3, 5, 7 using western blot (*n* = 3). The data were represented as mean ± SD. *p < 0.05, **p < 0.01, ***p < 0.001. ns, no significant difference.

To elucidate the regular function of *SQOR* in UC, we analyzed the *SQOR* protein expression in intestinal tissues. We found that *SQOR* was highly expressed in healthy mouse colonic tissues, including the apical region of colon crypts (Figure 1A), then we retrieved the Human Protein Atlas database and found that *SQOR* is highly abundant in normal human healthy colonic tissues, especially in intestinal epithelial cells (Figure S1A).

To define the potential role of *SQOR* in the progression of acute UC, we employed DSS‐induced UC models, administering 3% DSS in drinking water to WT mice. After DSS stimulating, the colitis mice exhibited damaged and absent crypt abscesses, severe colonic tissue damage, and downregulated *SQOR* expression (Figure 1A). The western blot also showed that the *SQOR* level was reduced after DSS treatment (Figure 1B,C). Furthermore, we found the colon substantially shortened and enteritis gradually worsened (Figure 1D,E). In addition, colitis mice showed a reduction in H_2_S levels in colonic tissue (Figure S1B). In WT mice, *SQOR* levels decreased during disease progression (Figure 1F,G). We explored *SQOR* enzyme activity as described by Marutani et al. [19, 21], and observed significantly inhibited *SQOR* enzyme activity in colonic tissues after DSS treatment (Figure S1C). Furthermore, we found that DSS treatment also induced a decrease in persulfide levels (Figure S1D). These results suggested that its potential is important in intestinal homeostasis and diseases.

### 2.2 *SQOR* Deficiency in the Intestinal Epithelial Cells Exacerbates DSS‐Induced UC

**FIGURE 2:**
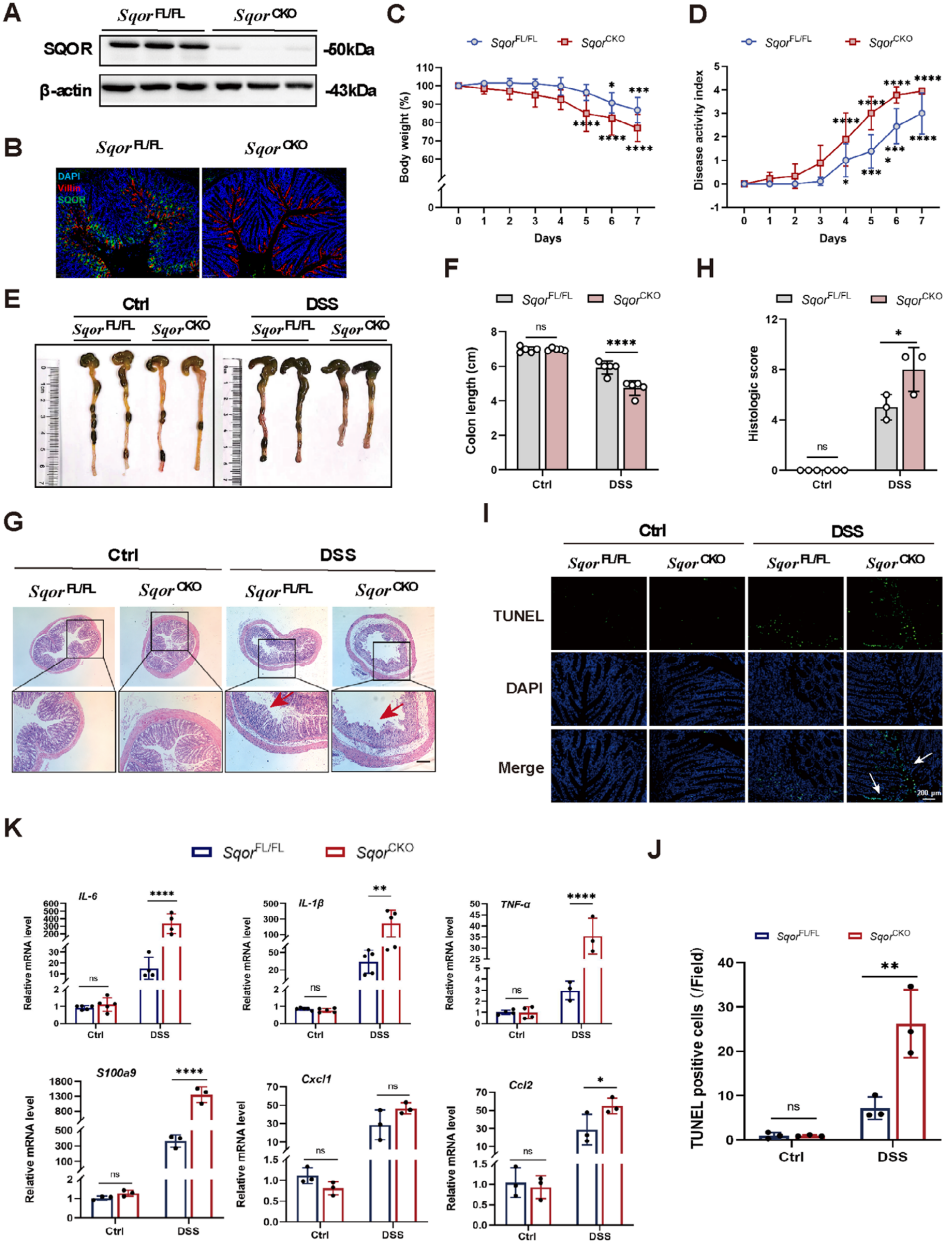
SQOR deficiency in the intestinal epithelial cells exacerbates DSS‐induced UC. (A) Western blot analysis of *SQOR* level in intestinal epithelial cells from *Sqor* ^FL/FL^ and *Sqor* ^CKO^ mice (*n* = 3). (B) Immunofluorescence analysis of *SQOR* (green), Villin (red), and DAPI (blue) in the mouse colonic section was determined by immunofluorescence staining (scale bar: 100 µm; *n* = 3). (C, D) Daily body weight changes and DAI of *Sqor* ^FL/FL^ and SQOR^CKO^ mice treated with 3% DSS. (E, F) A representative photograph of colon of *Sqor* ^FL/FL^ and *Sqor* ^CKO^ mice on day 7 after DSS treated or not, and the colon length was recorded. (G, H) The histological analysis of colon sections was performed H&E staining from *Sqor* ^FL/FL^ and SQOR^CKO^ mice after DSS treated or not (scale bar: 200 µm; enlarged scale bar: 100 µm), histological scores from *Sqor* ^FL/FL^ and *Sqor* ^CKO^ mice after DSS treated or not (*n* = 3). (I, J) Apoptotic cells in colonic sections as determined by TUNEL assay (scale bar: 200 µm; *n* = 3). (K) Cytokines and chemokines mRNA levels in colon tissues from *Sqor* ^FL/FL^ and *Sqor* ^CKO^ mice after DSS treated or not. The data were represented as mean ± SD. *n* = 6 mice per group. *p < 0.05, **p < 0.01, ***p < 0.001, ****p < 0.0001. ns, no significant difference.

To directly investigate *SQOR* in acute UC pathogenesis, we generated *Sqor* ^FL/FL^‐Villin Cre mice lacking *SQOR* specifically in intestinal epithelial cells (Figure S2A), and observed the depletion of *SQOR* in intestinal epithelial cells by western blot and immunofluorescence staining (Figure 2A,B). The body weights of *Sqor* ^CKO^ mice were not significantly different from *Sqor* ^FL/FL^ mice without DSS challenge (Figure S2B). When *Sqor* ^CKO^ mice and *Sqor* ^FL/FL^ mice were challenged with DSS, more severe colitis was observed in *Sqor* ^CKO^ mice than *Sqor* ^FL/FL^ mice, characterized by markedly body weight loss (Figure 2C), higher disease activity index (DAI) score (Figure 2D). Consistently, *Sqor* ^CKO^ mice also showed colon shortening in DSS‐induced mice (Figure 2E,F). The colonic morphology from *Sqor* ^FL/FL^ mice and *Sqor* ^CKO^ control mice demonstrated intact colonic morphology (Figure 2G). Histological evaluation showed that *SQOR* deficiency led to an increased score of crypt abscesses, crypt loss, and ulceration, with a significant increase in immune cell infiltration in response to DSS stimulation (Figure 2G,H). Additionally, TUNEL staining indicated that *Sqor* ^CKO^ mice exhibited enhanced cell death in the colon sections than *Sqor* ^FL/FL^ mice, up to 25%, with majority of cell death occurring in the intestinal epithelium (Figure 2I,J). In addition, we also determined the colonic H_2_S levels after DSS stimulation in *Sqor* ^FL/FL^ and *Sqor* ^CKO^ mice. As shown in Figure S2C, H_2_S levels in *Sqor* ^CKO^ colitis mice were significantly reduced.

During enteritis, significant infiltration of inflammation‐associated factors such as TNF‐α, IL‐1β, and IL‐6 causes damage to intestinal epithelial cells, triggering an increase in intestinal permeability. This pathological process reflects inflammatory factors in intestinal health and their potential influence on developing intestinal diseases. Quantitative analysis showed markedly elevated TNF‐α, IL‐1β, and IL‐6 expression in *Sqor* ^CKO^ mice than in *Sqor* ^FL/FL^ mice following DSS treatment (Figure 2K). Chemokines are critical cytokines in the pathogenesis of IBD. When chemokines bind to their specific receptors, they disrupt immune homeostasis in the gut, leading to the abnormal accumulation of inflammatory mediators and NF‐κB in the gut and exacerbating the inflammatory response. After DSS treatment, S100a9, Cxcl1, and Ccl2 expression levels in *Sqor* ^CKO^ mice were elevated compared to *Sqor* ^FL/FL^ mice (Figure 2K). It suggested that *SQOR* deficiency promoted colonic inflammation and aggravated colonic mucosal structure by DSS stimulation.

### 2.3 *SQOR* Deficiency Reduces Intestinal Barrier Function in DSS‐Induced Acute UC in Mice

**FIGURE 3:**
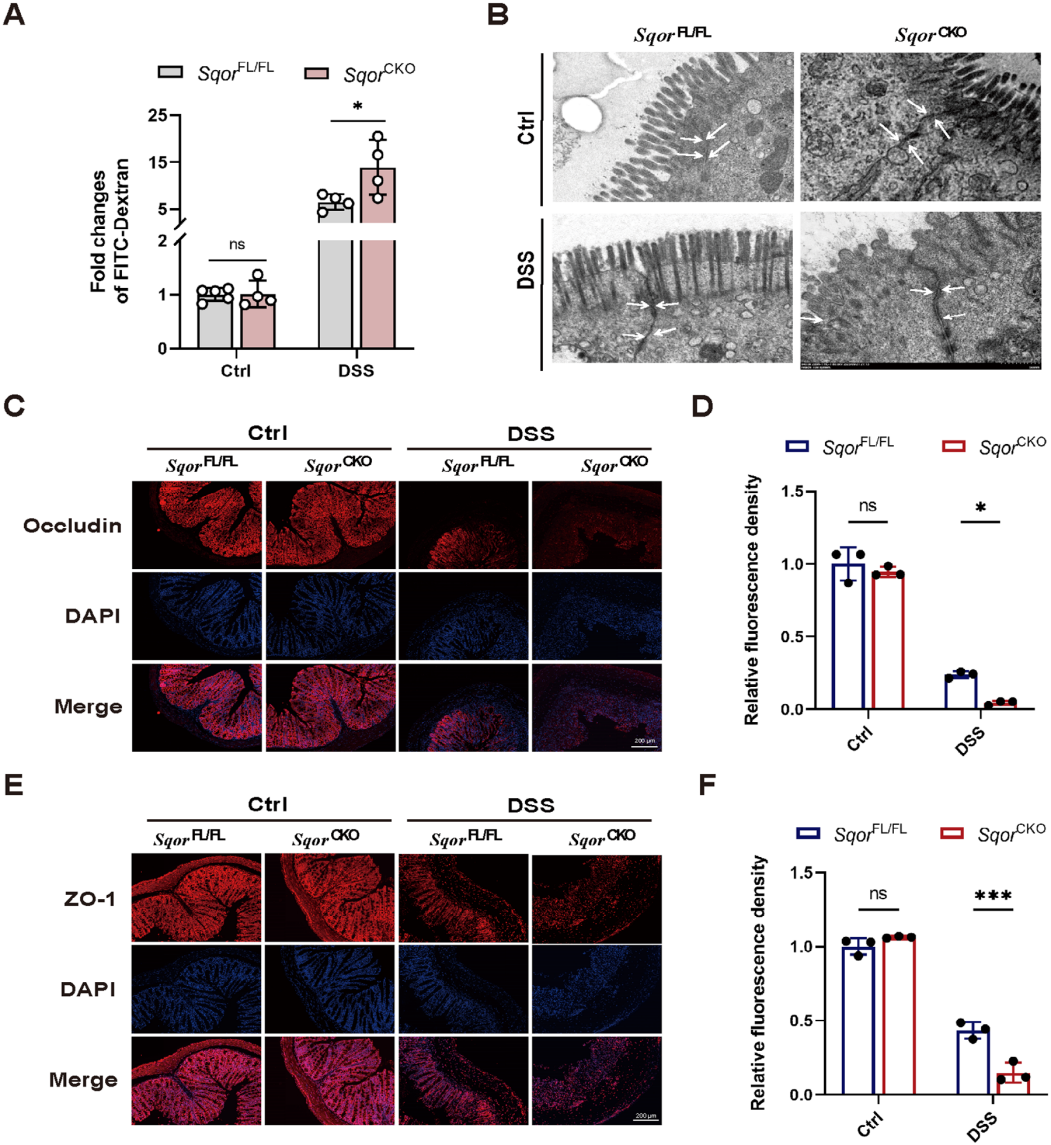
SQOR deficiency reduces intestinal barrier function in DSS‐induced acute UC in mice. (A) FITC‐dextran of serum determined intestinal permeability from *Sqor* ^FL/FL^ and *Sqor* ^CKO^ mice after DSS treated or not (*n* = 4). (B) Representative TEM images of tight junctions between intestinal epithelial cells from *Sqor* ^FL/FL^ and *Sqor* ^CKO^ mice after DSS treatment or not, white arrows indicating tight junctions. (C, D) The occludin in the mouse colon sections was determined by immunofluorescence staining from *Sqor* ^FL/FL^ and *Sqor* ^CKO^ mice after DSS treated or not (scale bar: 200 µm). (E, F) The ZO‐1 in the mouse colon sections was determined by immunofluorescence staining from *Sqor* ^FL/FL^ and *Sqor* ^CKO^ mice after DSS treated or not (scale bar: 200 µm). The data were represented as mean ± SD. *n* = 3 per group. *p < 0.05, **p < 0.01, ***p < 0.001. ns, no significant difference.

To investigate the pathogenesis related to *SQOR* on intestinal barrier function, we measured fluorescein isothiocyanate (FITC)‐dextran concentration in the serum of mice undergoing acute DSS‐induced colitis. Results showed that significantly increased vessel permeability in *Sqor* ^FL/FL^ colitis mice and *Sqor* ^CKO^ colitis mice, as compared with control mice (Figure 3A). Notably, FITC‐dextran concentration in *Sqor* ^CKO^ mice was higher than in *Sqor* ^FL/FL^ colitis mice (Figure 3A), indicating that *SQOR* deficiency increases intestinal permeability in DSS‐induced acute UC mice. Next, we assessed colonic mucosa by transmission electron microscopy (TEM), which showed that *Sqor* ^FL/FL^ mice and *Sqor* ^CKO^ mice had complete tight junctions without DSS treatment (Figure 3B). In contrast, the tight junctions of *Sqor* ^CKO^ mice were severely disrupted after DSS treatment (Figure 3B).

Consequently, immunofluorescence staining examined the colonic tight junction proteins occludin and zonula occludens‐1 (ZO‐1) expression levels. The levels of occludin and ZO‐1 were reduced in both groups of mice after DSS treatment, and the levels of occludin and ZO‐1 were significantly lower in *Sqor* ^CKO^ mice than in *Sqor* ^FL/FL^ mice (Figure 3C–F). Similarly, occludin and ZO‐1 mRNA levels were significantly reduced in *Sqor* ^CKO^ colitis mice (Figure S3A,B). These data demonstrate that *SQOR* deficiency exacerbated the DSS‐induced epithelial barrier dysfunction.

### 2.4 *SQOR* Deficiency Drives Mitochondrial Damage in Intestinal Epithelial Cells

**FIGURE 4:**
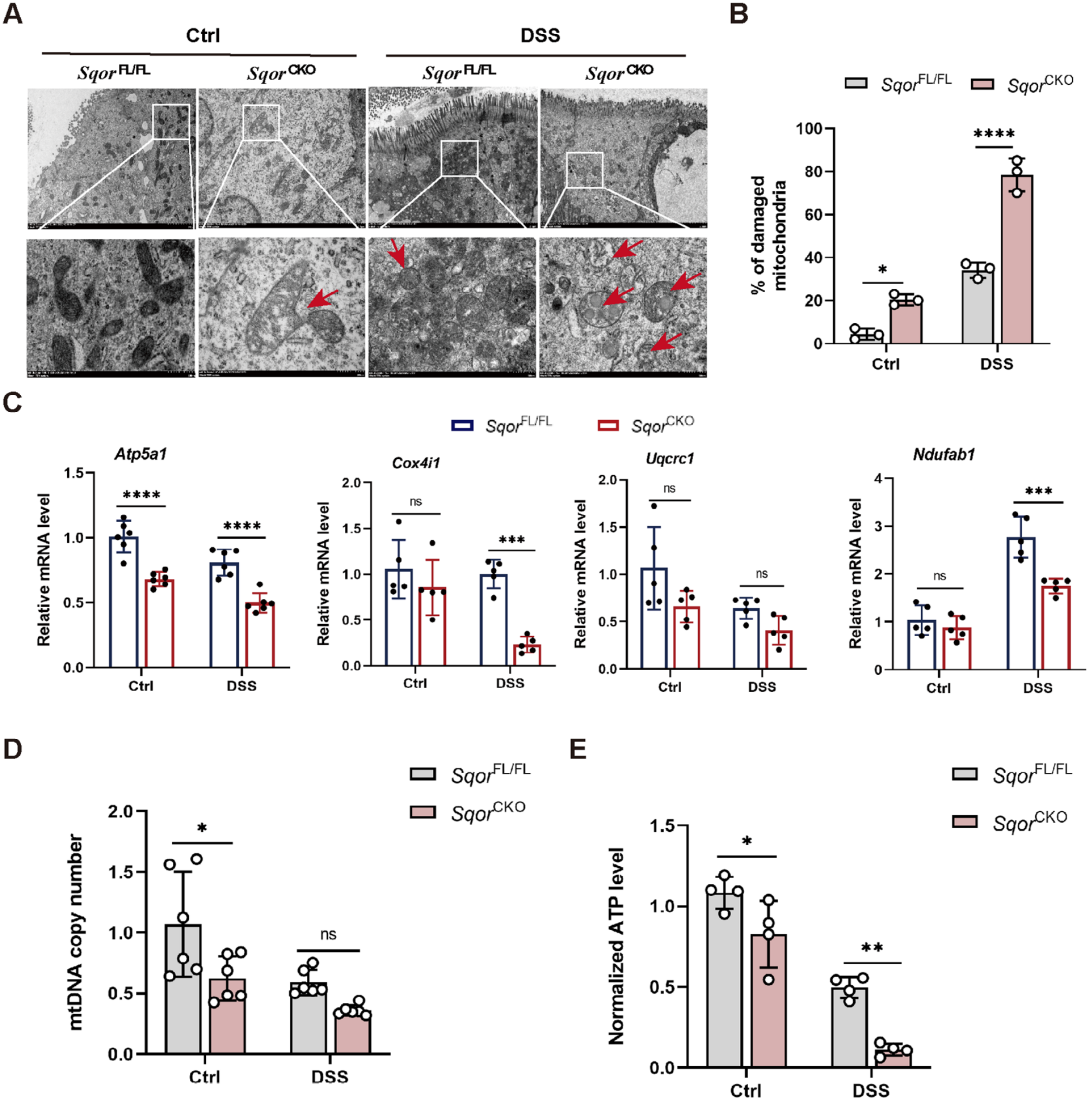
SQOR deficiency drives mitochondrial damage in intestinal epithelial cells. (A) Representative mitochondria images of DSS‐stimulated intestinal epithelial cells by TEM from *Sqor* ^FL/FL^ and *Sqor* ^CKO^ mice after DSS treated or not, red arrows indicate damaged mitochondria (scale bar: 5 µm; enlarged scale bar: 500 nm; *n* = 3). (B) Percentage of damaged mitochondria (*n* = 3). (C) Detection of the mRNA levels of Atp5a1, Cox4i1, Uqcrc1, and Ndufab1 in intestinal epithelial cells from *Sqor* ^FL/FL^ and *Sqor* ^CKO^ mice after DSS treated or not (*n* = 5). (D) The mtDNA copy number in intestinal epithelial cells from *Sqor* ^FL/FL^ and *Sqor* ^CKO^ mice after DSS treated or not (*n* = 6). (E) The ATP level in intestinal epithelial cells from *Sqor* ^FL/FL^ and *Sqor* ^CKO^ mice after DSS treated or not (*n* = 4). The data were represented as mean ± SD. *p < 0.05, **p < 0.01, ***p < 0.001, ****p < 0.0001. ns, no significant difference.

Previous studies revealed that *SQOR* is closely related to mitochondrial homeostasis and *SQOR* knockout exacerbates cisplatin‐induced mitochondrial dysfunction in renal tubular cells [14]. Accordingly, we used TEM to observe the mitochondrial morphology of intestinal epithelial cells. We observed that mitochondria from *Sqor* ^CKO^ mice reduced mitochondrial density and cristae fragmentation compared with intact mitochondrial structure in *Sqor* ^FL/FL^ mice without DSS challenge (Figure 4A). In addition, *SQOR* deficiency exacerbated mitochondrial damage, manifesting as swollen mitochondria and cristae breakage intensified, with a mitochondrial damage rate of up to 80% after DSS stimulation (Figure 4B).

The activity of the mitochondrial ETC complex is inextricably linked to mitochondrial function. ETC complex activity reduction results in a disruption of energy metabolism. It has been previously reported that *SQOR*‐mediated H_2_S oxidation is involved in mitochondrial ETC electron transport [22, 23]. Therefore, we investigated whether *SQOR* deficiency affected ETC complex expression. Next, we examined the levels of the mitochondrial electron transport chain complex relative genes, including Atp5a1, Cox4i1, Uqcrc1, and Ndufab1. However, the Atp5a1 was markedly decreased, and the levels of Cox4i1 and Uqcrc1 exhibited a slight reduction in *Sqor* ^CKO^ mice compared to *Sqor* ^FL/FL^ mice without DSS challenge (Figure 4C). Consistently, the mRNA expression of Atp5a1, Cox4i1, and Ndufab1 were significantly lower in the intestinal epithelial cells of *Sqor* ^CKO^ mice than in *Sqor* ^FL/FL^ mice after DSS treatment (Figure 4C).

Several studies have reported that reducing human mitochondrial DNA (mtDNA) in cells impaired mitochondrial function [24]. We found that the copy number of mtDNA in *Sqor* ^CKO^ mice that were not treated with DSS was significantly lower than that of SQOR^FL/FL^ mice, suggesting mitochondrial function was impaired in untreated *Sqor* ^CKO^ mice under normal conditions (Figure 4D). Furthermore, the mtDNA copy number was reduced in both mice following DSS stimulation, and there was lower in *Sqor* ^CKO^ mice than in *Sqor* ^FL/FL^ mice (Figure 4D).

The primary role of mitochondria drives ATP synthesis through oxidative phosphorylation, thereby meeting the majority of the cell energy requirements [25]. It has been demonstrated that the development of intestinal inflammation is associated with low levels of ATP in the mucosa. Consequently, we measured the ATP levels in intestinal epithelial cells. We found that ATP levels in the intestinal epithelial cells of *Sqor* ^CKO^ mice were markedly lower compared to *Sqor* ^FL/FL^ mice without or with DSS treated (Figure 4E). It indicated that the mitochondrial energy supply in the intestinal epithelial cells of *Sqor* ^CKO^ mice was compromised.

### 2.5 *SQOR* Maintains Mitochondrial Dynamics Homeostasis

To investigate how *SQOR*‐mediated protection against UC in the intestinal epithelial cells, we stimulated NCM460 cells with DSS to induce inflammatory responses. The results showed higher TNF‐α, IL‐6, and IL‐1β mRNA levels in response to different DSS concentrations (5, 10, 20 µg/mL) over 24 h (Figure S4A). Notably, the inflammation response of DSS on NCM460 cells was dose‐dependent (Figure S4A). Thus, 20 µg/mL DSS was selected for further experiments. At the same time, western blot and QPCR analysis demonstrated that siRNA successfully silenced *SQOR* expression (Figure S4B,C).

As a critical marker of mitochondrial function, mitochondrial membrane potential is often impaired in colitis, and studies showed that DSS‐induced mitochondrial impairment in mouse intestinal epithelial cells could be restored by increasing mitochondrial membrane potential and ATP levels [26]. Indeed, DSS treatment induced a loss of lower mitochondrial membrane potential. Strikingly, *SQOR* silence exacerbated mitochondrial dysfunction, characterized by lower mitochondrial membrane potential in DSS‐stimulated NCM460 cells (Figure S4D,E). The O_2_ consumption rate (OCR) is a crucial criterion for evaluating mitochondrial respiration, we found that *SQOR* silence could inhibit oxidative phosphorylation in NCM460 cells mitochondria (Figure S5A). It also reduced the basal respiratory capacity, maximal respiratory value and reserve respiratory capacity, slightly reducing the ATP production of NCM460 cells (Figure S5B–E).

**FIGURE 5:**
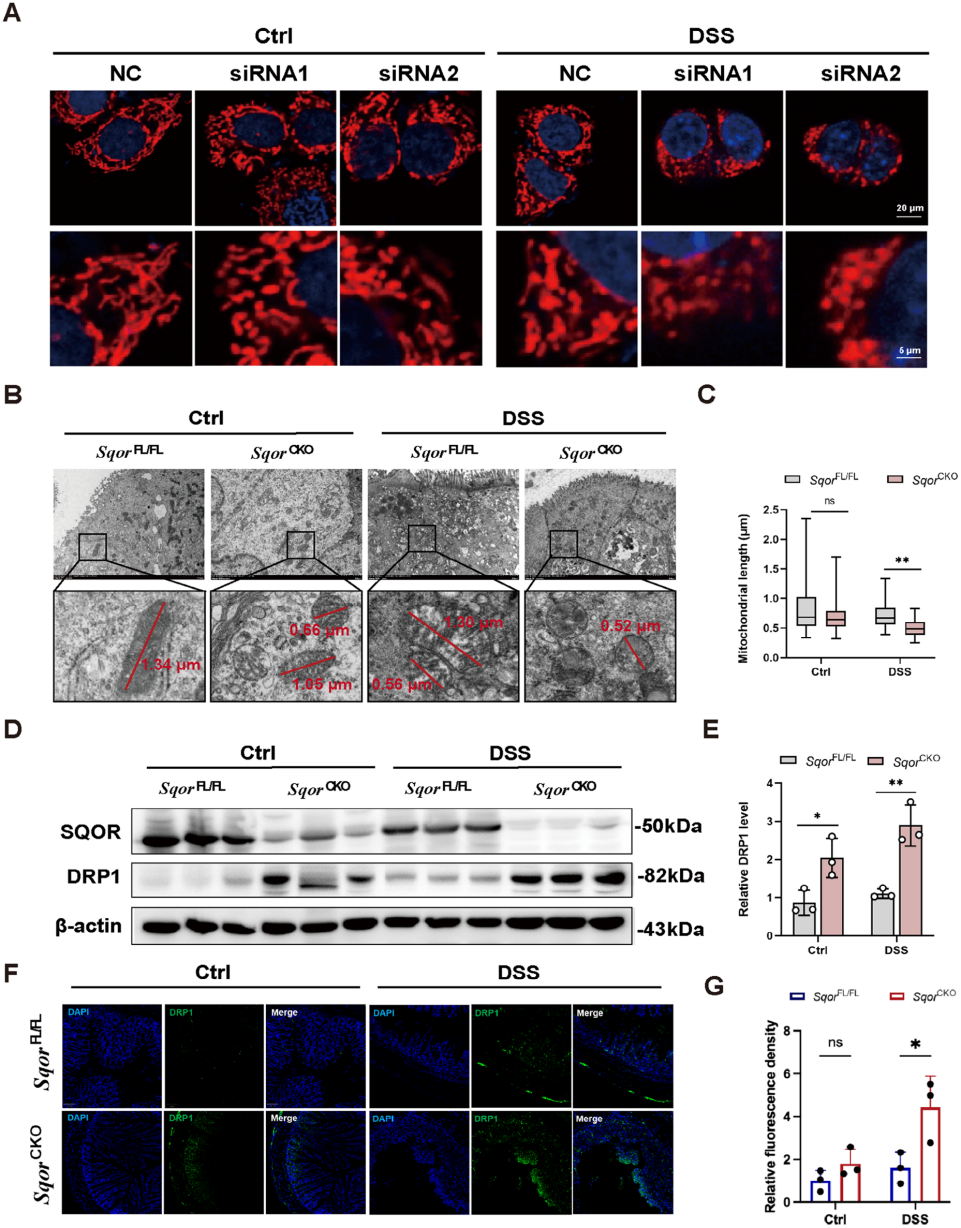
SQOR maintains mitochondrial dynamics homeostasis. (A) Mito‐Tracker was employed to mark mitochondria in NCM460 cells with transfected siRNA‐*SQOR* or NC siRNA in the presence or absence of DSS (scale bar: 20 µm; enlarged scale bar: 5 µm). (B) Representative TEM of mitochondria images in intestinal epithelial cells from *Sqor* ^FL/FL^ and *Sqor* ^CKO^ mice after DSS treated or not, red lines represent mitochondrial length measurements. (C) Quantitative analysis of mitochondrial length in TEM images. (D, E) Western blot analysis of *SQOR* and DRP1 in intestinal epithelial cells from *Sqor* ^FL/FL^ and *Sqor* ^CKO^ mice after DSS treated or not. (F, G) The DRP1 in the mouse colon sections was determined by immunofluorescence staining from *Sqor* ^FL/FL^ and *Sqor* ^CKO^ mice after DSS treated or not (scale bar: 100 µm). The data were represented as mean ± SD. *n* = 3 per group. *p < 0.05, **p < 0.01. ns, no significant difference.

Any imbalance in mitochondrial division may affect mitochondrial function, which triggers disturbance in energy metabolism, exacerbating ROS overproduction and activating inflammation signaling pathways [27, 28]. We evaluated the mitochondrial morphology to determine whether the increased severity of colitis observed in *Sqor* ^CKO^ mice relative to *Sqor* ^FL/FL^ mice was driven by mitochondrial dysfunction. We observed that silencing *SQOR* increased punctate mitochondria compared to typical interconnected filamentous and reticular structures in NCM460 cells (Figure 5A). Moreover, it showed a marked increase in the punctate distribution of mitochondria, disrupting filamentous and reticular structures in *SQOR*‐silenced groups compared to controls after the DSS challenge (Figure 5A). TEM analysis characterized the mitochondrial morphology of intestinal epithelial cells in *Sqor* ^FL/FL^ mice as having a long rod and globular mitochondria, with an average mitochondrial length of 0.80 µm. At the same time, *Sqor* ^CKO^ mice showed reduced rod mitochondria, increased mitochondria, and an average mitochondrial length of 0.69 µm (Figure 5B,C). After DSS stimulation, most of the mitochondria in *Sqor* ^CKO^ intestinal epithelial cells were globular, with the average length decreasing to 0.51 µm, which was significantly lower than the average length of mitochondria in the intestinal epithelial cells of *Sqor* ^FL/FL^ mice (Figure 5B,C).

Next, we will address whether *SQOR* influences mitochondrial division. DRP1, as a core mitochondrial division protein, was significantly higher in the intestinal epithelial cells of *Sqor* ^CKO^ mice than those of *Sqor* ^FL/FL^ mice without DSS treatment, indicating that *SQOR* deficiency promoted excessive mitochondrial division in intestinal epithelial cells (Figure 5D,E). The DRP1 protein in DSS‐stimulated *Sqor* ^CKO^ mice was also increased compared with that in *Sqor* ^FL/FL^ mice (Figure 5D,E). Drp1 immunofluorescence data also corroborated increased presence in *Sqor* ^CKO^ mice after stimulation (Figure 5F,G). Furthermore, we used the mitochondrial division inhibitor 1 (Mdivi‐1), a selective inhibitor of Drp1‐treated DSS‐induced UC mice. It found that *Sqor* ^CKO^ mice treated with Mdivi‐1 slightly improved the symptoms of colitis mice, which is evidenced by a relative reduction in fecal blood occurrence and relative long in colon length compared with only DSS‐treated *Sqor* ^CKO^ mice (Figure S6). Together, our data demonstrated that loss of *SQOR* disrupts mitochondrial function in intestinal epithelial cells by promoting excessive mitochondrial division.

### 2.6 Intestinal Epithelial Cell Function Is Associated With ROS

**FIGURE 6:**
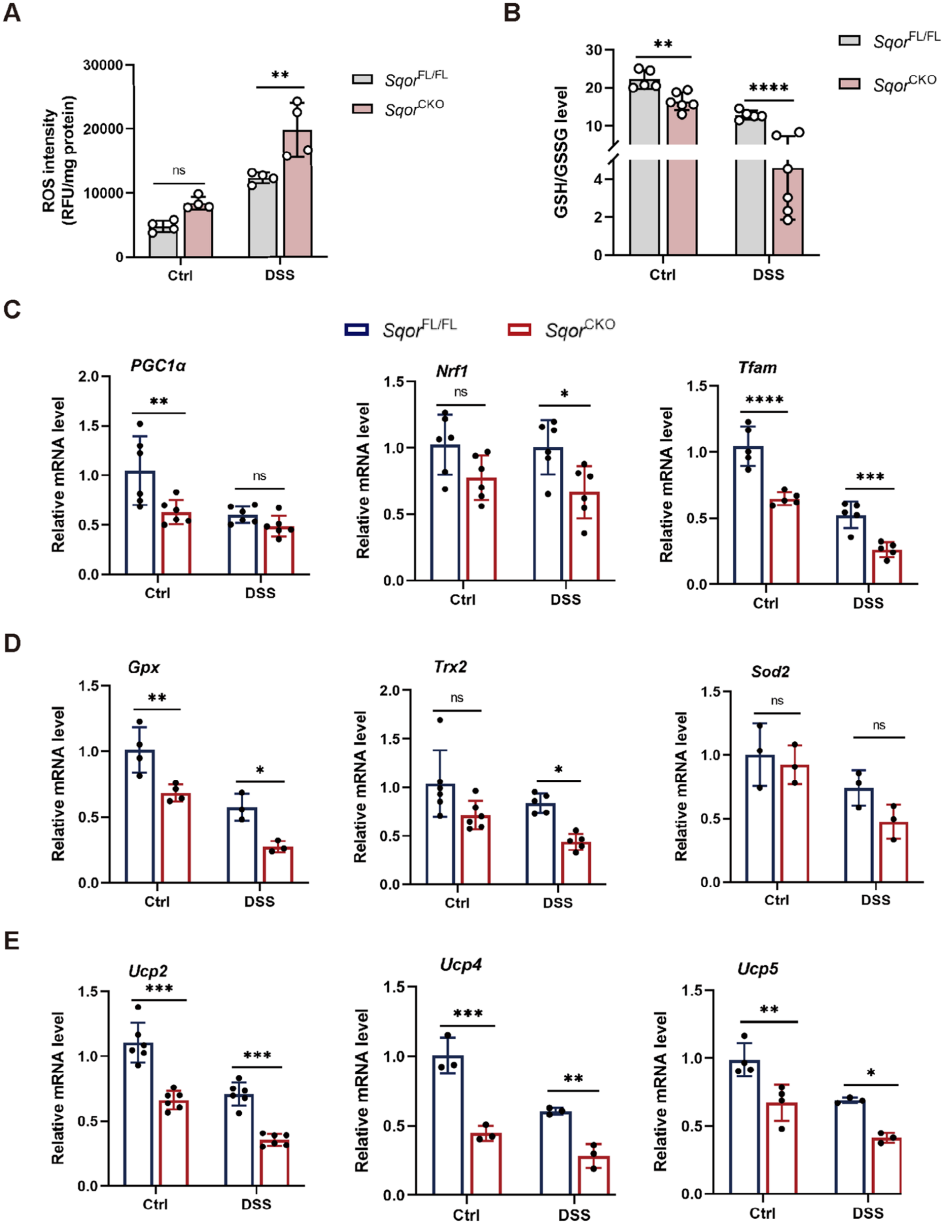
Intestinal epithelial cell function is associated with ROS. (A) Detection of ROS level in intestinal epithelial cells from *Sqor* ^FL/FL^ and *Sqor* ^CKO^ mice after DSS treated or not (*n* = 4). (B) GSH/GSSG ratio in intestinal epithelial cells from *Sqor* ^FL/FL^ and *Sqor* ^CKO^ mice after DSS treated or not (*n* = 6). (C) The mRNA levels of PGC1α, Nrf1, and Tfam in mouse intestinal epithelial cells from *Sqor* ^FL/FL^ and *Sqor* ^CKO^ mice after DSS treated or not (*n* = 6). (D) The mRNA levels of Gpx, Trx2, and Sod2 in mouse intestinal epithelial cells from *Sqor* ^FL/FL^ and *Sqor* ^CKO^ mice after DSS treated or not (*n* = 4). (E) The mRNA levels of Ucp2, Ucp4, and Ucp5 in mouse intestinal epithelial cells from *Sqor* ^FL/FL^ and *Sqor* ^CKO^ mice after DSS treated or not (*n* = 3). The data were represented as mean ± SD. *n* = 6 per group. *p < 0.05, **p < 0.01, ***p < 0.001. ns, not significant.

Alternation in mitochondrial dynamics, particularly fission processes, may induce ROS generation to initiate redox signaling [29], we explored whether *SQOR* affects ROS levels in intestinal epithelial cells. Silencing *SQOR* significantly increased ROS levels without DSS stimulation compared to the control group. When *SQOR* knockdown cells treated with DSS resulted in markedly increased ROS levels in NCM460 cells (Figure S7A,B). Similar observations were made in the colitis mouse model, compared to *Sqor* ^FL/FL^ colitis mice, ROS levels in *Sqor* ^CKO^ colitis mice were significantly elevated in the intestinal epithelial cells (Figure 6A), which may be associated with excessive mitochondrial division. It suggested that *SQOR* maintains cellular redox levels by regulating mitochondrial dynamics and inhibiting ROS generation in intestinal epithelial cells.

**FIGURE 7:**
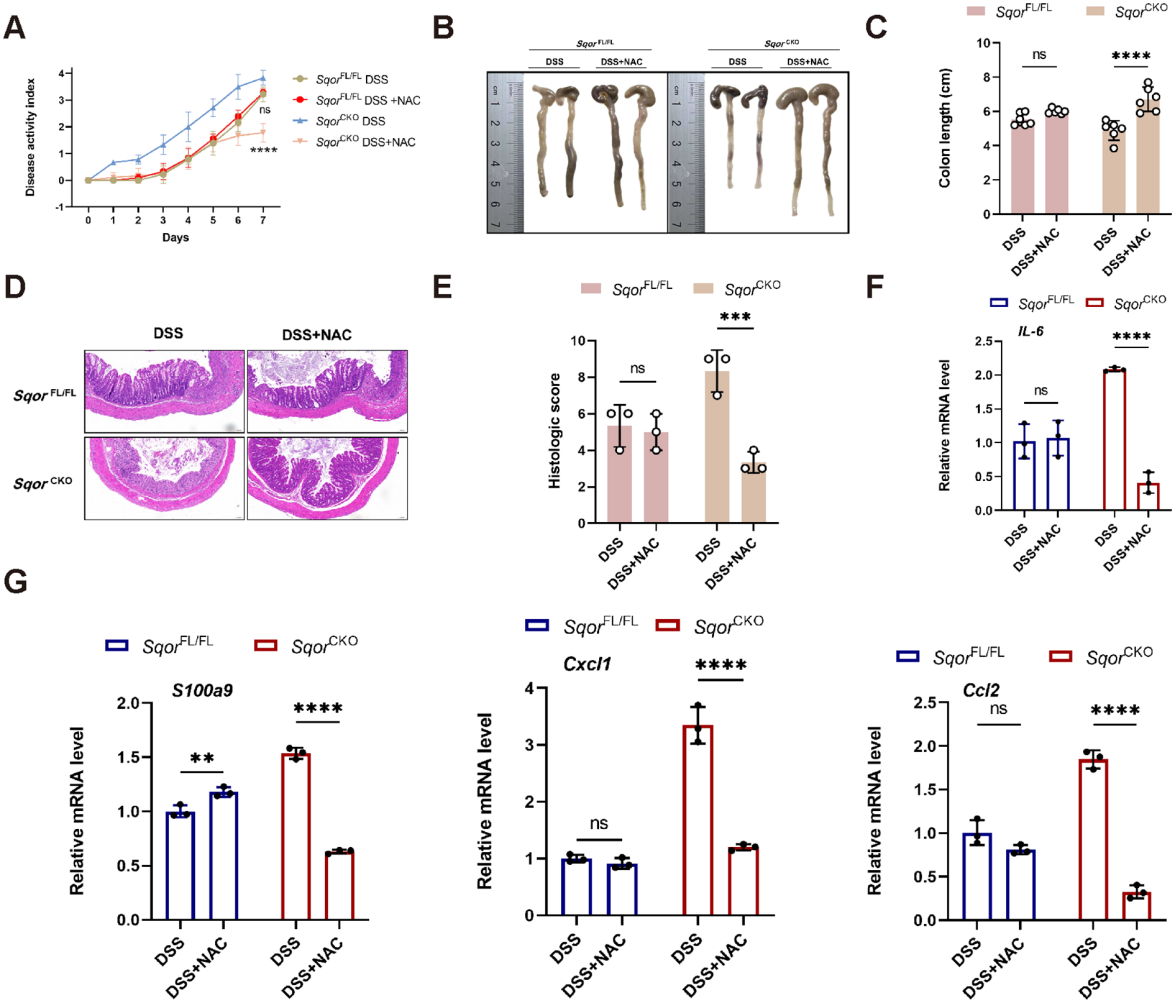
NAC alleviates DSS‐induced colitis model. (A) Daily DAI of *Sqor* ^FL/FL^ and *Sqor* ^CKO^ colitis mice treated with NAC. (B, C) A representative photograph of colon of *Sqor* ^FL/FL^ and *Sqor* ^CKO^ mice colitis mice treated with NAC, and the colon length was recorded. (D, E) The histological analysis of colon sections was performed H&E staining from *Sqor* ^FL/FL^ and *Sqor* ^CKO^ mice treated with NAC (scale bar: 100 µm), histological scores from *Sqor* ^FL/FL^ and *Sqor* ^CKO^ mice treated with NAC (*n* = 3). (F, G) Cytokines and chemokines mRNA levels in colon tissues from *Sqor* ^FL/FL^ and *Sqor* ^CKO^ mice treated with NAC (*n* = 3). The data were represented as mean ± SD. *n* = 6 mice per group. *p < 0.05, **p < 0.01, ***p < 0.001, ****p < 0.0001. ns, no significant difference.

We thus tested whether ROS scavenger acetyl cysteine (NAC) can alleviate DSS‐induced colitis in mice. Acute colitis was induced with DSS and then administered additional NAC to mice. The NAC rescued the severe spontaneous colitis phenotypes of *Sqor* ^CKO^ colitis mice, as evidenced by lower DAI and longer colon length (Figure 7A–C). NAC treatment also remarkably decreased histological scores, with a significant decrease in immune cell infiltration in response to DSS stimulation (Figure 7D,E), which is consistent with observed reduced inflammatory cytokine expression including IL‐6, S100a9, cxcl1, and ccl2 in colonic tissues of *Sqor* ^CKO^ mice (Figure 7F,G). Therefore, *SQOR* regulated intestinal epithelial cell function via intracellular ROS.

Reduced GSH could scavenge ROS, and the GSH/GSSG ratio is a well‐established indicator of cellular redox status [30]. GSH downregulation has been reported in inflamed intestinal tissues of IBD patients and a DSS‐induced mouse model of colitis [31]. Next, we examined the GSH/GSSG levels in intestinal epithelial cells. Compared with *Sqor* ^FL/FL^ mice, *Sqor* ^CKO^ mice had significantly lower GSH/GSSG without DSS treatment (Figure 6B). Indeed, DSS treatment induced markedly reduced GSH/GSSG levels in *Sqor* ^CKO^ mice (Figure 6B). Malondialdehyde (MDA) assays also revealed *Sqor* ^CKO^ colitis mice had markedly increased mitochondria lipid peroxidation (Figure S8A). It has been reported that GPX4 is one of the master suppressor of ferroptosis [32]. We first examined GPX4 expression in *Sqor* ^FL/FL^ and *Sqor* ^CKO^ mice colonic sections by immunofluorescence staining. GPX4 showed a decreasing trend in *Sqor* ^CKO^ mice that were not treated with DSS. After DSS was treated the GPX4 was markedly reduced in *Sqor* ^FL/FL^ and *Sqor* ^CKO^ mice, and there was a lower in *Sqor* ^CKO^ mice than in *Sqor* ^FL/FL^ mice (Figure S8B,C). *Sqor* ^CKO^ mice also markedly reduced the expression of ferroptosis‐related genes, including Gpx4 and Fsp1, slightly reduced Slc7a11 expression (Figure S8D). Thus, *SQOR* as modulator of tolerance of intestinal epithelial cells to oxidative damage and ferroptosis.

Although the IBD pathogenesis is multifactorial, growing evidence implicates that redox imbalance in intestinal epithelial cells and aberrant mitochondrial bioenergetics are important causative factors in the development of the disease [33]. PGC1α is a significant regulator of mitochondrial biosynthesis, oxidative phosphorylation, and oxidative stress responses [34]. Studies reported that the levels of PGC1α are closely correlated with the number of mitochondria, and PGC1α deficiency leads to mitochondrial dysfunction, resulting in metabolic disorders, and ultimately cell death [35, 36]. PGC1α and Nrf1 interaction upregulates mtDNA expression and modulates the expression of mitochondrial antioxidant genes [37]. Tfam binds to and entangles mtDNA and maintains mtDNA structure, transcription, and replication [38]. Notably, immunofluorescence analysis confirmed the downregulated expression of PGC1α in colonic sections in *Sqor* ^CKO^ mice treated with DSS (Figure S9).

Furthermore, oxidative‐related genes PGC1α, Nrf1, and Tfam in *Sqor* ^CKO^ mice were significantly lower than those in *Sqor* ^FL/FL^ mice with or without DSS treatment, suggesting that less *SQOR* inhibited mitochondrial biogenesis in intestinal epithelial cells, and the ability of mitochondria to resist oxidative damage decreased (Figure 6C). PGC1α can regulate the expression of mitochondrial antioxidant genes (Gpx, Trx2, and Sod2) and uncoupling protein genes (Ucp2, Ucp4, and Ucp5) to scavenge ROS [37]. The expression of these mitochondrial antioxidant and uncoupling protein genes in *Sqor* ^CKO^ mice was lower than that in *Sqor* ^FL/FL^ mice with or without DSS treatment, suggesting that *SQOR* knockout reduced the ROS scavenging ability of intestinal epithelial cells (Figure 6D,E).

## 3 Discussion

IBD represents chronic inflammatory disorder characterized by relapsing and remitting inflammation symptoms including CD and UC. Recent advances in IBD therapeutics highlight promising agents under investigation, including Janus kinase (JAK) inhibitors, cytokine inhibitors (IL‐12/IL‐23), TNF‐like cytokine 1A (TL1A) inhibitors, and fatty acid modulators [39]. Although these biologics and small molecule drugs have provided promising benefits to IBD patients, there is still an urgent requirement for effective therapeutic.

**FIGURE 8:**
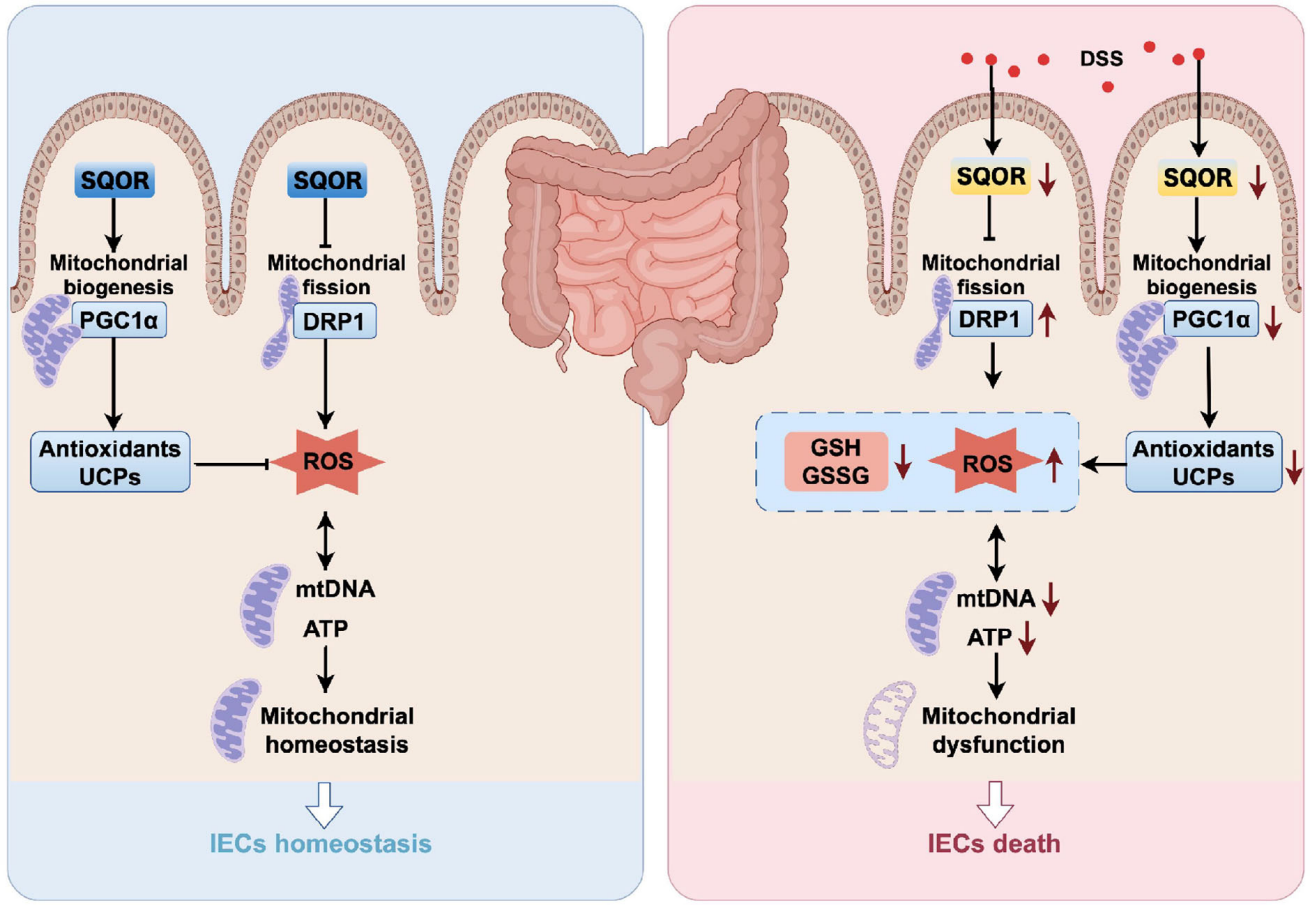
A proposed mechanism by which SQOR alleviated DSS‐induced acute UC by ameliorating mitochondrial dysfunction.

*SQOR* initiates irreversible oxidation of H_2_S, which is in mitochondria [23]. Recently, studies revealed that *SQOR* is involved in the H_2_S‐mediated mitochondrial homeostasis and ganoderic acids biosynthesis in *Ganoderma lucidum* under heat stress [40]. Deletion of *SQOR* abolishes therapeutic benefits of H_2_S following intracerebral hemorrhage by targeting mitochondrial electron transport chain function to induce mitochondrial uncoupling [23]. Here, we generated *Sqor* ^FL/FL^‐Villin Cre mice, which lack *SQOR* specifically in intestinal epithelial cells, to investigate whether *SQOR* regulates of the occurrence and development of enteritis. It was found that *SQOR* deficiency drives severe DSS‐induced acute colitis. We also identified the effect of *SQOR*, which may rely on it to regulate ROS levels in intestinal epithelial cells by excessive mitochondrial division inhibition and mitochondrial biogenesis induction (Figure 8).

Intestinal barrier dysregulation may cause pathological responses to tissue damage and microbiota, which are associated with disease severity in IBD intestinal fibrosis, and colorectal cancer [41, 42]. A hallmark of the gut in patients with IBD is characterized by increased epithelial tight junction permeability [43, 44]. Leakage of bacteria due to intestinal permeability increases can make the harmful substances and macromolecules enter the body through the intestinal wall, thereby exacerbating enteritis [45]. Mirroring our experimental findings in control versus colitis mice, epithelial dysregulation characterized intestinal disease. Here, we found that *SQOR* could attenuate DSS‐driven compromise of colonic barrier function and tight junction integrity.

Accumulating data support that mitochondrial activity of respiratory chain complexes II, III, and IV decreased by approximately 50%–60% in UC colonic mucosa [46], but the molecular mechanism behind this was unclear [18]. Altered mitochondrial function in intestinal epithelial cells contributes to the development of IBD. This finding has inspired more profound research into mitochondria's critical role in maintaining intestinal health [47]. Also, we observed through TEM that part of the mitochondrial ridge was broken or disappeared in the intestinal epithelial cells of *Sqor* ^CKO^ mice without DSS treatment. Silencing *SQOR* leads to mitochondrial dysfunction and further aggravates the acute colitis induced by DSS in mice. Thus, *SQOR* was further established as a regulator of colitis that functions in the intestinal epithelial cells.

Human mtDNA is a cyclic molecule comprising 16,568 bases, which encode ribosomal RNA, transfer RNA, and essential components of mitochondrial ETC [48, 49]. Considering that a reduction in the number of mtDNAs in cells has been demonstrated to impair mitochondrial function [24], we hypothesized that less *SQOR*‐induced mitochondrial dysfunction in damaged intestinal epithelial cells involves mtDNA. Notably, previous studies showed that the mitochondrial genome encoding the 13 genes that regulate ATP production was significantly reduced in patients with UC. The rate‐limiting step in oxidative phosphorylation also reduced the mitochondrial electron transport chain complex I activity [10, 50, 51]. Consistent with our findings, deletion of *SQOR* leads to mitochondrial damage, decreased mtDNA copy number and ATP levels in intestinal epithelial cells. Hence, *SQOR* in intestinal epithelial cells plays a prominent role in regulating mitochondrial function.

Most products of mitochondrial respiration are reaction oxygen species (ROS), which facilitate Ca^2+^‐dependent mitochondrial permeability transition (MPT)‐regulated cell death [52]. Furthermore, mitochondrial ROS (mtROS) could trigger inflammation by driving proinflammation cytokine production [53]. Therefore, the balance of mtROS is necessary to maintain intestinal epithelial homeostasis [54]. Any imbalance in mitochondrial division may affect mitochondrial function, which triggers disturbances in energy metabolism, exacerbates the overproduction of ROS, and activates inflammation signaling pathways. Together, these chain reactions exacerbate mitochondrial dysfunction, posing a severe threat to cellular health [28]. Notably, earlier work has already revealed that overproduction of ROS occurs in both human IBD colonic tissue and experimental colitis models [46]. Based on these, we found that *SQOR* deficiency promoted the increase of ROS levels in intestinal epithelial cells of colitis mice. Consistently, we found that ROS scavenger acetyl cysteine (NAC) alleviates DSS‐induced colitis in *Sqor^CKO^* mice. Therefore, *SQOR* regulates cytokines and chemokines expression involved in the ROS‐dependence inflammation in colonic epithelial cells, which are probably responsible for controlling colitis [55]. Accordingly, recent study showed that intestinal‐specific mice partial inhibition of mitochondrial ATP synthase attenuates inflammation through mitochondrial mtROS‐dependent NF‐kB activation [53].

Studies have shown that excessive mitochondrial division can produce large number of ROS, causing oxidative stress in cells. Although mtROS are an essential component of host defense and the immune system [56], high levels of ROS under pathological conditions can catalyze iron‐dependent Fenton reactions, generating free radicals that destroy macromolecules within mitochondria and oxidize mtDNA [57]. Because of its proximity to the ETC (a source of mtROS), mtDNA is susceptible to oxidative damage from mtROS. In this case, ROS are the drivers of the inflammatory response [58]. Increased ROS production happened in the colonic mucosa of patients with active UC [59], and excess ROS may trigger an imbalanced environment, leading to oxidative damage, inflammatory responses, and apoptosis in intestinal tissues [60]. We observed that silencing *SQOR* inhibited mitochondrial biogenesis in intestinal epithelial cells, and the ability of mitochondria to resist oxidative damage decreased. Furthermore, *SQOR* increased the expression of mitochondrial antioxidant genes and uncoupling protein genes by promoting mitochondrial biogenesis, increasing the ROS scavenging capacity. However, we tried the *SQOR* rescue experiment, but it was ineffective, which may be *SQOR* was highly expressed in healthy mouse colonic tissues. A limitation including whether the detection of *SQOR* can be used as a prognostic tool requires future studies.

In summary, our study demonstrated that *SQOR* was downregulated during colitis and *SQOR* deficiency in the intestine aggravates DSS‐induced UC. Most importantly, *SQOR* regulates ROS levels in intestinal epithelial cells by inhibiting excessive mitochondrial division, promoting mitochondrial biogenesis, and maintaining mitochondrial function homeostasis. Collectively, these results indicated that *SQOR* may help protect against UC associated with mitochondrial dysfunction.

## 4 Materials and Methods

### 4.1 Cell Culture

NCM460, a normal human colon mucosal epithelial cell line, was purchased from the American Type Culture Collection (ATCC, USA), and maintained in our laboratory. NCM460 cells were maintained in DMEM supplemented with 10% fetal bovine serum (FBS, SenBeiJia, China), 100 U/mL penicillin, and 100 µg/mL streptomycin in a humidified atmosphere of 5% CO_2_ at 37°C.

### 4.2 Animals

C57BL/6 mice (6–8 weeks) were purchased from the Cavens (Changzhou, China). To generate the *Sqor* ^CKO^ mouse, we crossed commercially available mice carrying *Sqor* ^Flox/Flox^ (*Sqor* ^FL/FL^) with mice with villin‐cre transgenic mice, the *Sqor* ^FL/FL^ littermates were used as WT mice. Genotyping was performed using PCR amplification and then PCR product was analyzed by gel electrophoresis. The genotyping primers were listed in Table S1. All mice were housed in a temperature‐controlled sterile room. The Nanjing University Animal Care and Use Committee approved all animal experiments.

### 4.3 Animal Model and Treatment

*Sqor* ^CKO^ mice and *Sqor* ^FL/FL^ mice were treated with sterile water containing 3% (w/v) DSS for 7 days to induce UC mice. The control group mice drink water at the same time. The DAI was determined by scoring changes in body weight, blood in stool, and stool consistency. The body weight of mice was recorded daily.

Intestinal permeability was assessed by orally administrating FITC‐dextran. Briefly, following a 4 h fasting period, mice received 50 mg/mL FITC‐dextran (Sigma‐Aldrich, St Louis, MO, USA). After 4 h, serum samples were collected to determine FITC‐dextran concentration via a fluorescence reader (Bio‐Tek, USA).

Mdivi‐1 (HY‐15886, MCE) and ROS scavenger acetyl cysteine (NAC, HY‐B0215, MCE) were used in DSS‐induced UC treatment. Mdivi‐1 and NAC were dissolved according to the manufacturer's instructions. To be treated with Mdivi‐1 in vivo, mice received daily intraperitoneal injections of Mdivi‐1 (25 mg/kg) for 7 days. To be treated with NAC in vivo, mice received daily intraperitoneal injections of NAC (100 mg/kg) once daily for 7 days. The control mice were injected intraperitoneally with vehicle solution.

### 4.4 H_2_S Measurements

The colon is lysed and then centrifuged at 12,000 rpm for 10 min at 4°C. The lysate was added to a 96‐well plate, lined with lead acetate paper, and incubated at 37°C.

### 4.5 Enzyme Activity of *SQOR*

Colonic *SQOR* activity was assessed as reported previously with modification [19]. The colon was lysed using RIPA buffer, then the lysates were centrifuged at 12,000 rpm for 10 min at 4°C. The protein was concentrated to 10 mg/mL, then diluted 1:20 in PBS containing 22.2 µM coenzyme Q10. Dissolved Na_2_S in 0.2 M Tris–HCl buffer. And diluted supernatant was combined with Na_2_S solution in the cuvette.

### 4.6 Intracellular Persulfide Measurement

Intracellular persulfide levels in NCM460 cells were quantified using sulfane sulfur probe 4 (SSP4, Dojindo, Japan) following the manufacturer's protocol. Briefly, cells were incubated with 20 µM SSP4 for 15 min in a humidified incubator, washed with PBS twice, and measured fluorescent intensity using a microplate reader.

### 4.7 Histology and Immunostaining

Colon samples were fixed 4% paraformaldehyde, paraffin‐embedded then stained with hematoxylin and eosin (H&E). Colonic tight junctions were stained by deparaffinization of 5 µm paraffin‐embedded sections. Primary antibodies were incubated overnight. After washing, sections were incubated for 1 h at room temperature with species‐matched secondary antibodies in the dark. Samples were analyzed with a sliced scanner. Cell death was quantified by TUNEL assay (Vazyme, China) following the manufacturer's protocols. The scores were the assessment of inflammatory cell infiltration and tissue damage.

### 4.8 Western Blot Assay

The colon was lysed using RIPA buffer (Beyotime Biotechnology, China) containing a protease inhibitor cocktail (Roche, Switzerland). Then, lysates were centrifuged at 12,000 rpm for 10 min at 4°C to remove cell remnants. After denaturation, the protein samples were separated on 12% SDS‐PAGE polyacrylamide gels and subsequently transferred to polyvinylidene difluoride (PVDF) membrane (Bio‐Rad, China). Protein‐loaded membranes were blocked with 5% (w/v) nonfat milk for 1 h, followed by overnight incubated with primary antibody: SQOR (Abcam, dilution 1:1000) at 4°C, washed three times with PBST and continuously incubated with the secondary antibody (dilution 1:2000) for 1 h at room temperature. Visualized protein bands were using enhanced chemiluminescence (ECL, Tannon, Shanghai, China). The relative band density was determined using Image J software.

### 4.9 RNA Isolation and RT‐PCR

Total RNA was isolated using the TRIzol reagent (Vazyme, China) following the manufacturer's protocol. One microgram of total RNA was reverse transcribed to cDNA using ReverTra Ace qPCR RT Kit (TOYOBO, Japan). Real‐time qPCR was performed on a StepOne Real‐Time PCR system with AceQ qPCR SYBR Green Master Mix (Vazyme, China) with β‐actin normalization. Primer sequences were listed in Table S2.

### 4.10 Transmission Electron Microscope

Fresh colon tissue was rinsed clean using cold PBS buffer and then cut into rings of about 2 mm in size. Then, the colon was fixed in 2.5% glutaraldehyde (Servicebio Biological Technology Co., China) at room temperature for 2 h. Subsequently, samples were pre‐embedded in 1% agarose gel, postfixed with 1% osmium tetroxide for 1 h, dehydrated through a series ethanol series and finally infiltrated with SPI‐Pon 812 resin mixture. Colonic tissue samples were reoriented to obtain horizontal sections. Thin sections (60–80 nm) were placed on gold support grids and contrasted with lead citrate and uranyl acetate. Images were acquired with a TEM (Hitachi, Japan).

### 4.11 Mitochondrial DNA Copy Number Assay

Total DNA was extracted from intestinal epithelial cells. mtDNA copy number was determined COX2 mRNA and normalized using nuclear Rps18 gene copies.

### 4.12 ATP Assay

Freshly intestinal epithelial cells were lysate in lysis buffer. Then they centrifuged for 10 min at 12,000 × *g*, and supernatants were immediately aliquoted for ATP measurement by ATP determination kit (Beyotime Biotechnology, China) according to the manufacturer's protocol.

### 4.13 Cell Transfection

SiRNA‐*SQOR* and negative control (NC) siRNA were purchased from Genepharma (Shanghai, China). The sequences were listed as siRNA‐*SQOR1* 5′‐GGUCGAAUUAUUCAGUUAATT‐3′, siRNA‐*SQOR2* 5′‐CGGAACCUCACUGUUAACUTT‐3′, si‐NC 5′‐UUCUCCGAACGUGUCACGUTT‐3′. Transfection was carried out using Lipofectamine 2000 (Invitrogen, USA) following the manufacturer's protocol.

### 4.14 Mitochondrial Function Assays

NCM460 cells were washed with PBS and labeled at 37°C for 30 min with Mito‐Tracker Red CMXRos (Beyotime Biotechnology, China) or TMRM probe (mitochondrial membrane potential). Fluorescence was detected on a confocal microscope (Carl Zeiss AG, Germany). Flow cytometry analysis detected mitochondrial membrane potential (NovoCyte, ACEA, Bioscience Inc, USA). Data collection and analysis were done using the NovoExpress software (Agilent Technologies, USA) following the manufacturer's instructions.

### 4.15 ROS Assay

Intestinal epithelial cells were lysate in lysis buffer and incubated with O13 (Bestbio, China) working solutions for 20 min at 37°C in the dark. The ROS levels were determined by the ROS kit (Bestbio, China) according to the manufacturer's protocol. NCM460 cells were incubated with a DCFH probe (1:1000, Beyotime Biotechnology, China), and flow cytometry analysis detected the ROS levels (NovoCyte, ACEA, Bioscience Inc, USA).

### 4.16 GSH/GSSG Assay

Freshly intestinal epithelial cells were added to protein removal reagent M solution, followed by two rapid freeze–thaw cycles using liquid nitrogen and a 37°C water bath. Centrifuge for 10 min at 10,000 × *g* and supernatants were immediately collected for GSH and GSSG levels using the GSH and GSSG Assay Kit (Beyotime Biotechnology, China).

### 4.17 Isolation of Colonic Epithelial Cells

Colonic tissues were gently flushed with cold PBS and cut into fragments, followed by incubation DMEM containing 8% FBS and DTE dithioerythritol for 20 min at 37°C. Epithelial cells were released by vigorous shaking, filtrated through a 70 µm filter, and collected by centrifugation at 2000 rpm for 10 min.

### 4.18 Seahorse Assays

OCR was determined using the Seahorse XFe96 Bioanalyzer (Seahorse Bioscience, MA, USA) following the manufacturer's instructions. In brief, for OCR determination, NCM460 cells were seeded in a Seahorse 96‐well plate, 1 h before measurement, growth the medium was replaced with prewarmed Seahorse XF medium supplemented with 1 mM sodium pyruvate, 2 mM glutamate, and 25 mM glucose were added. OCR was measured periodically at baseline and after sequential administration of oligomycin (1 µM), trifluoromethoxy carbonylcyanide phenylhydrazone (FCCP, 0.5 µM), and rotenone (Rot, 0.5 µM) and antimycin A (AMA, 0.5 µM; Rot&AMA).

### 4.19 Statistical Analysis

Statistical analyses were performed using GraphPad Prism (version 9) software. Data were expressed as means ± SD. Differences in the two groups were analyzed using Student's *t*‐test, and a one‐way analysis of variance (ANOVA) was performed to compare more than two groups. Data from multiple groups were analyzed using two‐way ANOVA. Differences between the groups were considered significant at *p* < 0.05.

## Author Contributions

Hongqin Zhuang, Zi‐Chun Hua, and Jian Cheng designed the outline of the paper. Hongqin Zhuang, Shuilian Fu, and Hailin Ma wrote the manuscript. Hailin Ma and Shuilian Fu performed most experiments in this study and prepared the figures. Chujun Huang, Na Han, and Fangfang Cai performed the experiments with the animals. Dangran Li and Fangfang Cai helped with the western bolt experiments. Shuilian Fu and Hailin Ma analyzed data. All the authors have read and approved the final version of this manuscript.

## Ethics Statement

All experiments involving animals were conducted according to the ethical policies and procedures approved by Nanjing University (Approval No: IACUC‐2102010).

## Conflicts of Interest

The authors declare no conflicts of interest.

## Supporting information



Supporting Information

## Data Availability

The data that support the findings of this study are available from the corresponding author upon reasonable request.
