# Supplementary material for: Sulfide Quinone Oxidoreductase Alleviates Acute Ulcerative Colitis by Regulating Mitochondrial Dysfunction
Source: MedComm (2020). 2025 Jul 13;6(7):e70285. doi: 10.1002/mco2.70285 (PMC12256674; doi:10.1002/mco2.70285)
Supplement: Supplementary file 1 — Supporting Information [file MCO2-6-e70285-s001.docx]

**Sulfide quinone oxidoreductase alleviates acute** **ulcerative colitis by regulating mitochondrial dysfunction**

Hailin Ma^1,#^, Shuilian Fu^1,#^, Chujun Huang^1^, Na Han^1^, Fangfang Cai^1^, Dangran Li^1^, Jian Cheng^4,*^, Hongqin Zhuang^1,*^, Zi-Chun Hua^1,2,3,*^

^1^The State Key Laboratory of Pharmaceutical Biotechnology, College of Life Sciences, Nanjing University, Nanjing, PR China

^2^Changzhou High-Tech Research Institute of Nanjing University and Jiangsu Target Pharma Laboratories Inc., Changzhou 213164, P. R. China

^3^Faculty of Pharmaceutical Sciences, Xinxiang Medical University, Xinxiang 453003, China

^4^Jiangsu Key Laboratory of Neuropsychiatric Diseases & Institute of Neuroscience, Soochow University, Suzhou, PR China

***Corresponding authors:**

Hongqin Zhuang, School of Life Sciences, Nanjing University, 163 Xianlin Blvd., Nanjing 210023, China. Phone: 86-25-89683692; Fax: 86-25-83324605, E-mail: [hqzhuang@nju.edu.cn](mailto:hqzhuang@nju.edu.cn)

Zi-Chun Hua, School of Life Sciences, Nanjing University, 163 Xianlin Blvd., Nanjing 210023, China. Phone: 86-25-89683692; Fax: 86-25-83324605, E-mail: hzc1117@nju.edu.cn

Jian Cheng, Institute of Neuroscience, Soochow University, 199 Renai Road, Suzhou, Jiangsu Province 215123, PR China. E-mail: [jiancheng8@hotmail.com](mailto:jiancheng8@hotmail.com)

**#These authors Contributed equally.**

**Supplementary Figures**

**
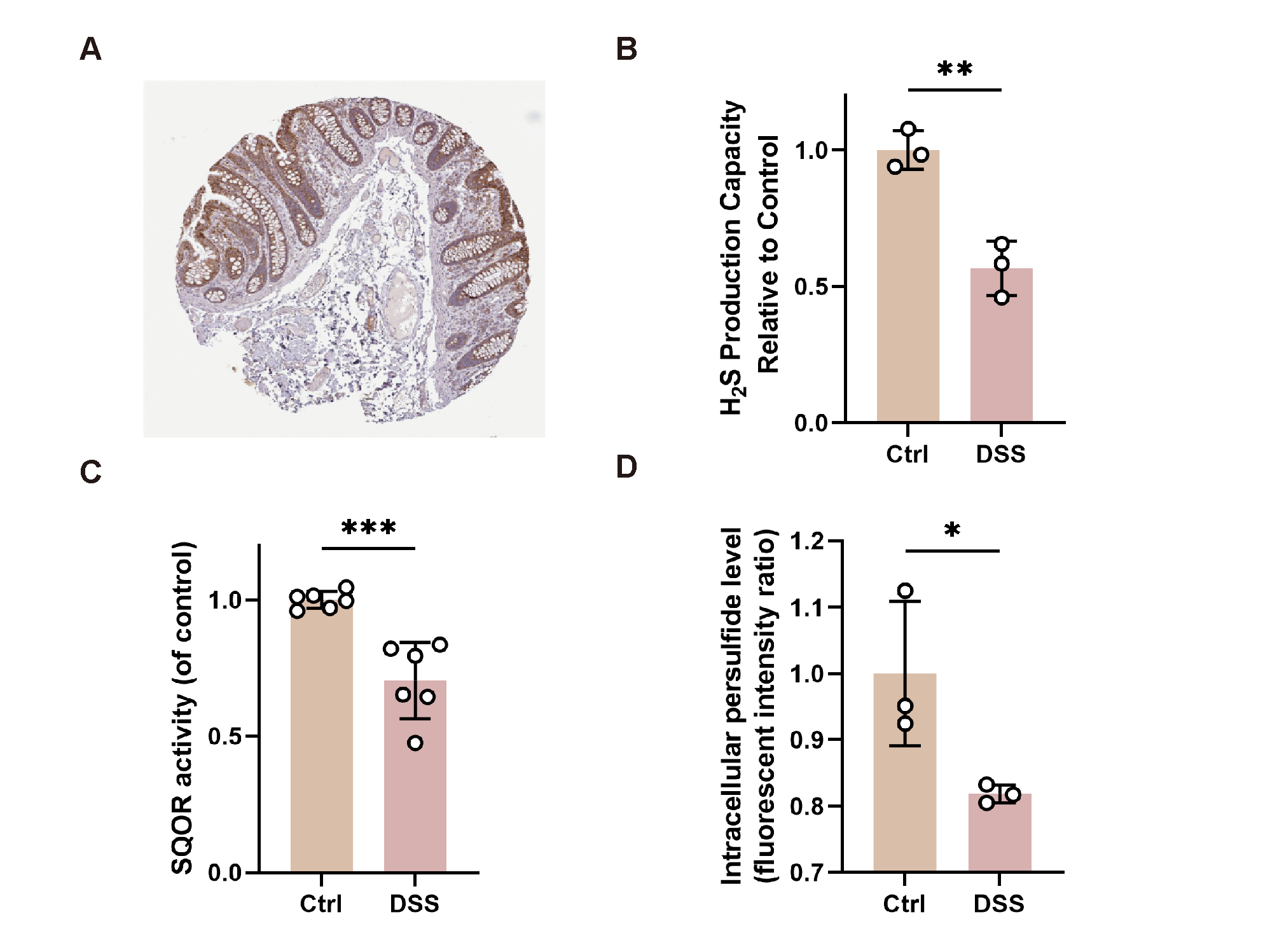
**

**Figure S1 DSS-induced acute UC significantly reduced SQOR levels.** (A) Representative IHC staining of SQOR in human normal colonic tissue. (B) Hydrogen sulfide levels in the colon of 3% DSS-treated mice (n=3). (C) The SQOR activity were measured in the colon tissues from 3% DSS-treated mice (n=6). (D) Intracellular persulfide levels were measured in DSS-treated NCM460 cells (n=3). The data were represented as mean ±SD. **P < 0.05, **P < 0.01, ***P < 0.001*.


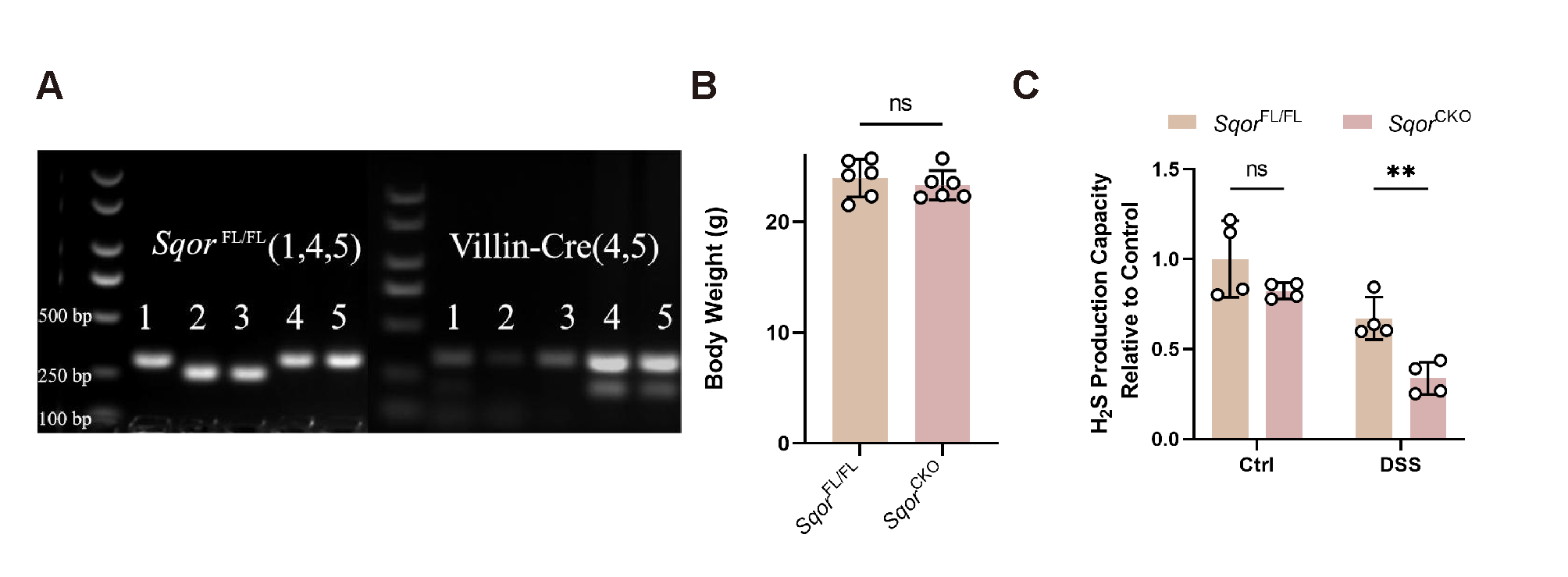


**Figure S2** **SQOR deficiency in** **the** **intestinal epithelial cells exacerbates DSS-induced UC.** (A) Representative DNA gel image of *Sqor*^FL/FL^ and *Sqor*^CKO^ mice. (B) Body weights of *Sqor*^FL/FL^ mice and *Sqor*^CKO^ mice without DSS challenge (n=6). (C) Hydrogen sulfide levels in the colonic tissues from *Sqor*^FL/FL^ and *Sqor*^CKO^ mice after DSS treated or not (n=4). The data were represented as mean ±SD. ***P < 0.01.* ns, no significant difference.


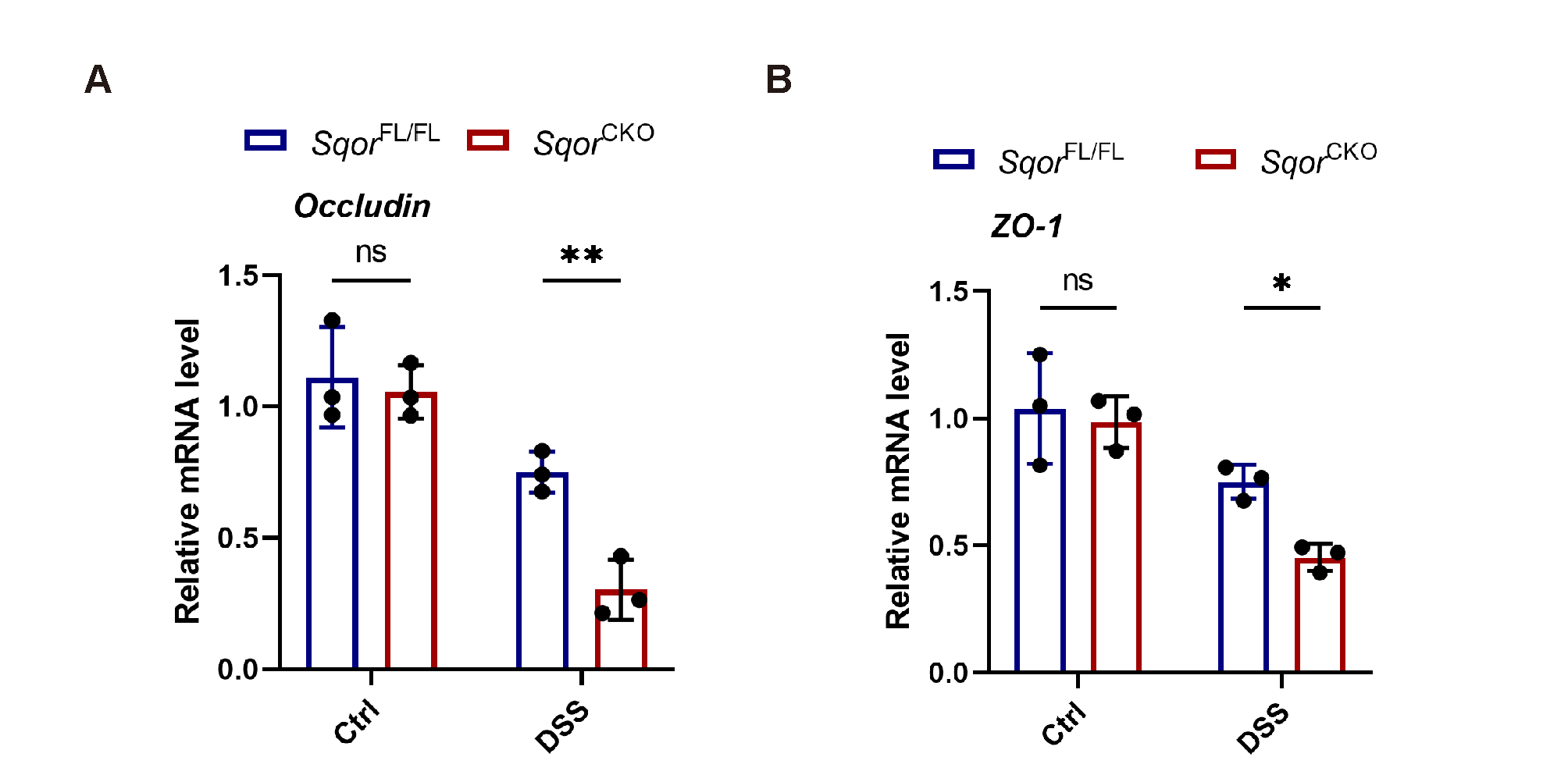


**Figure S3. SQOR deficiency reduces intestinal barrier function in DSS-induced acute UC in mice.** (A) The occludin mRNA levels in the mouse colon were determined from *Sqor*^FL/FL^ and *Sqor*^CKO^ mice after DSS treated or not. (B) The ZO-1 mRNA levels in the mouse colon were determined from *Sqor*^FL/FL^ and *Sqor*^CKO^ mice after DSS treated or not. The data were represented as mean ±SD. n=3 per group. **P < 0.05, **P < 0.01.* ns, no significant difference.


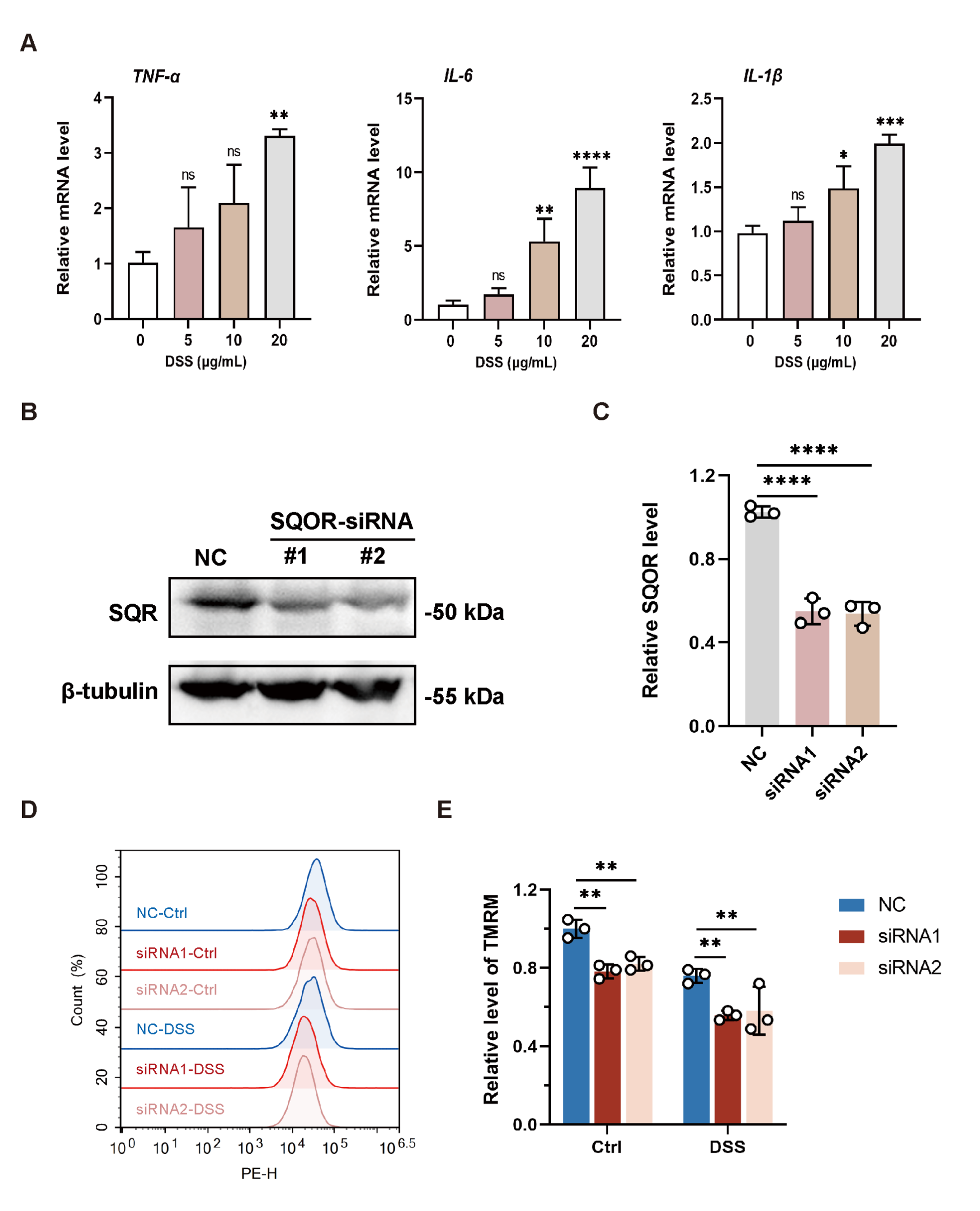


**Figure S4 SQOR maintains mitochondrial dynamics homeostasis.** (A) The mRNA levels of TNF-α, IL-6, IL-1β in DSS stimulated NCM460 cells. (B-C) NCM460 cells were transfected with SQOR siRNA or negative control siRNA for 48 h and then cells were prepared to detect SQOR expression using western blot. (D-E) Mitochondrial membrane potential detection by the TMRM in NCM460 cells with transfected with SQOR siRNA or negative control siRNA in the presence or absence of DSS. The data were represented as mean ±SD. n=3 per group. **P < 0.05, **P < 0.01, ***P < 0.001*. ns, no significant difference.


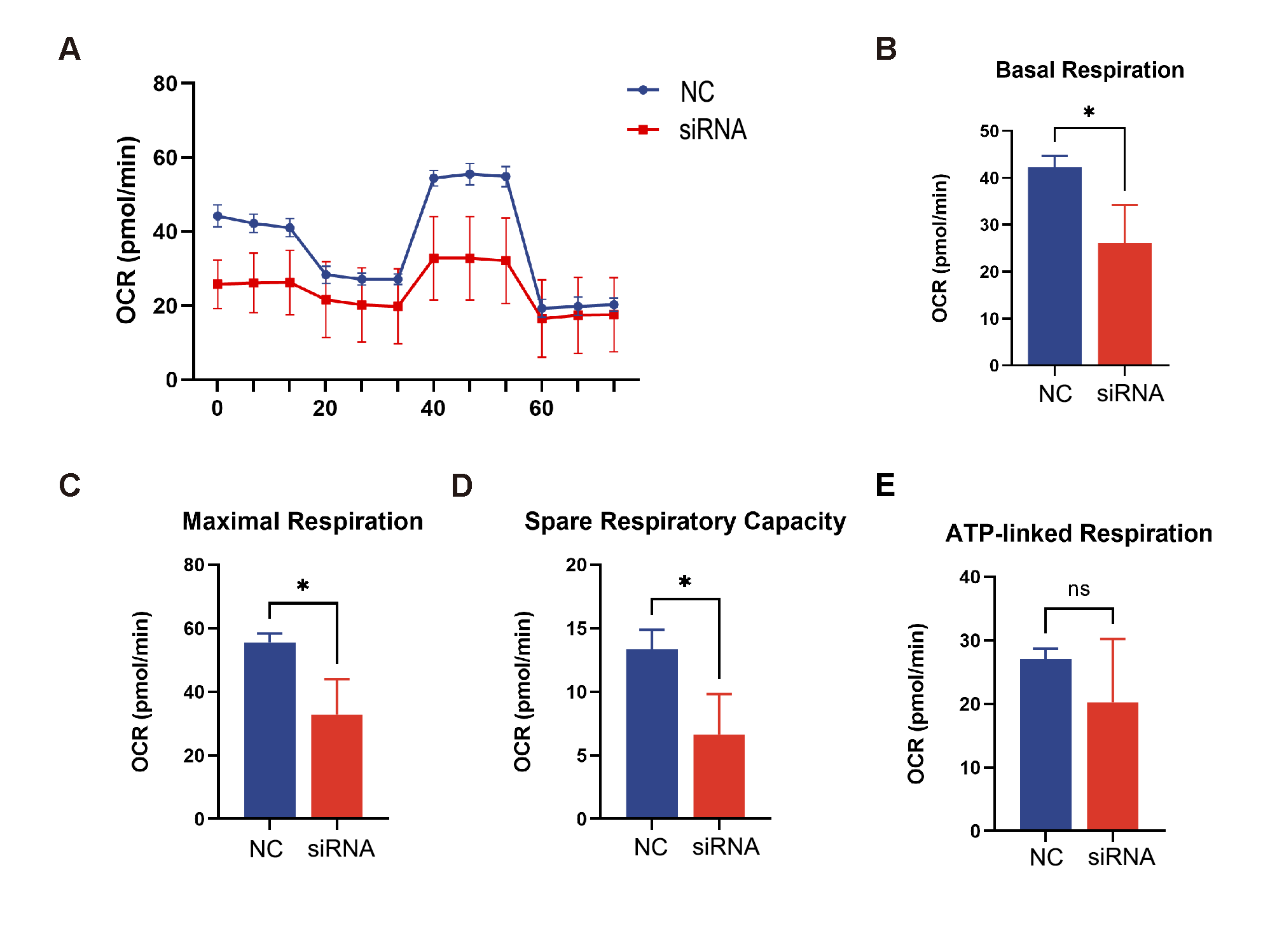


**Figure S5 SQOR maintains mitochondrial dynamics homeostasis.** (A)Seahorse analyses for OCR in NCM460 cells with transfected with SQOR siRNA or negative control siRNA in the presence or absence of DSS and quantitative results (B-E) of the different groups’ cellular OCR, basal, and maximal respiration rates. Basal respiration (B), Maximal respiration (C), Spare respiratory capacity (D), and ATP production (E). The data were represented as mean±SD. n=3 per group. *P < 0.05. ns, no significant difference.


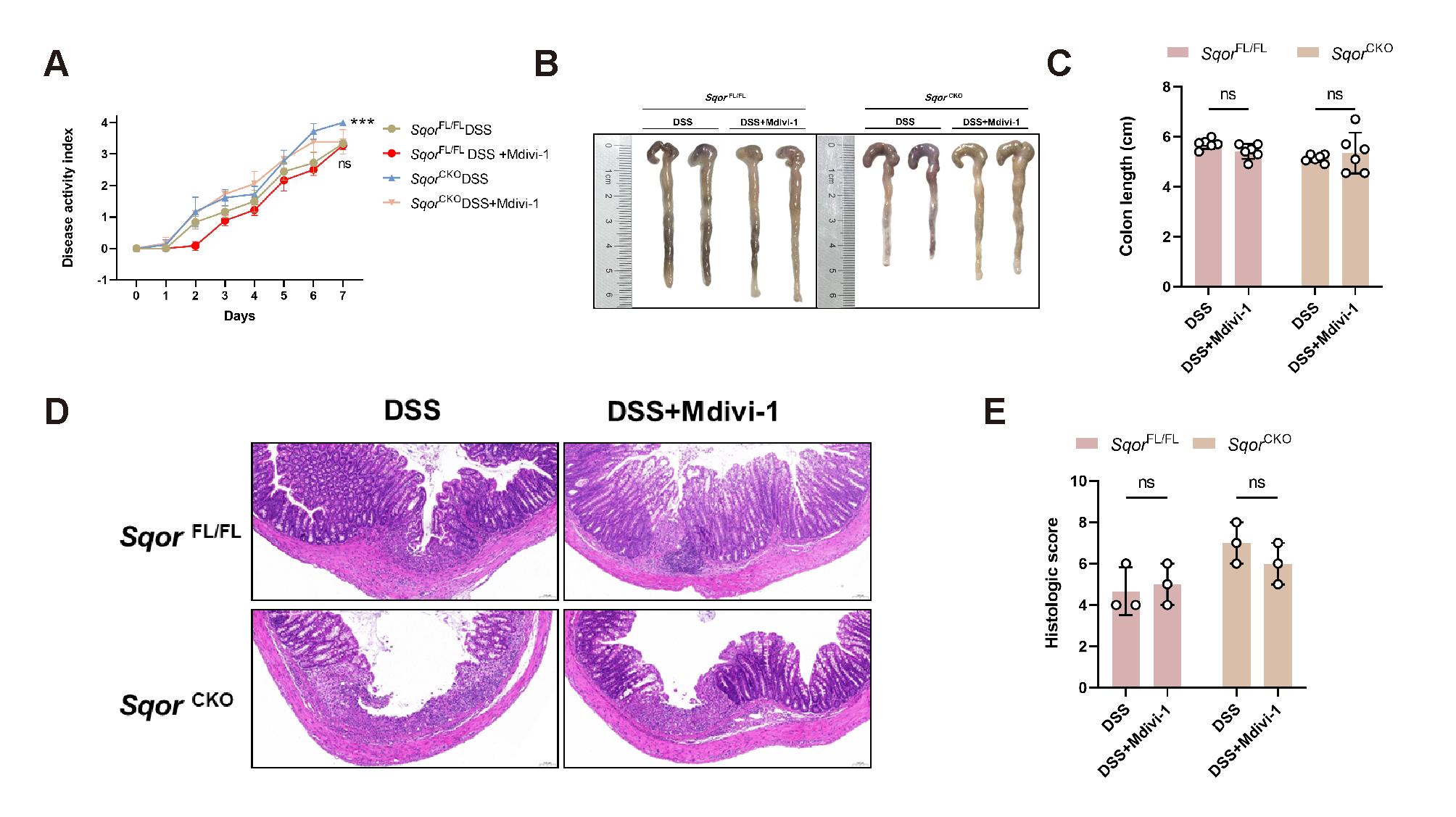


**Figure S6** **SQOR maintains mitochondrial dynamics homeostasis.** (A) Daily DAI of *Sqor*^FL/FL^ and *Sqor*^CKO^ colitis mice treated with Mdivi-1. (B-C) A representative photograph of colon of *Sqor*^FL/FL^ and *Sqor*^CKO^ mice colitis mice treated with Mdivi-1, and the colon length was recorded. (D-E) The histological analysis of colon sections was performed H&E staining from *Sqor*^FL/FL^ and *Sqor*^CKO^ mice a treated with Mdivi-1 (scale bar: 100 μm), histological scores from *Sqor*^FL/FL^ and *Sqor*^CKO^ mice treated with Mdivi-1 (n=3). The data were represented as mean ±SD. n=6 mice per group. ns, no significant difference.

**
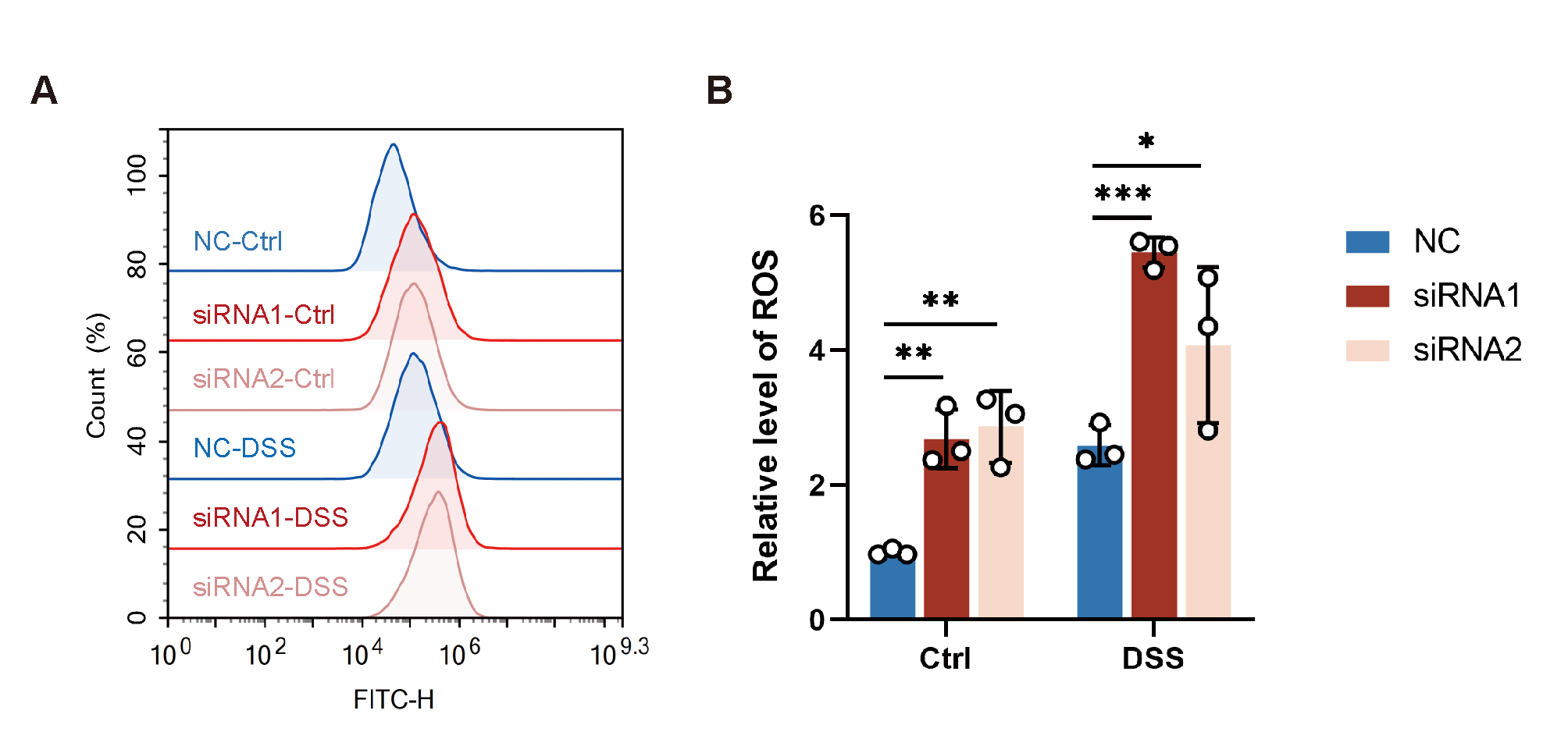
**

**Figure S7 Intestinal epithelial cells function is associate with ROS.** (A-B) Detection of ROS level in NCM460 cells with transfected with SQOR siRNA or negative control siRNA in the presence or absence of DSS. The data were represented as mean ±SD. n=3 per group. **P < 0.05, **P < 0.01, ***P < 0.001*.

**
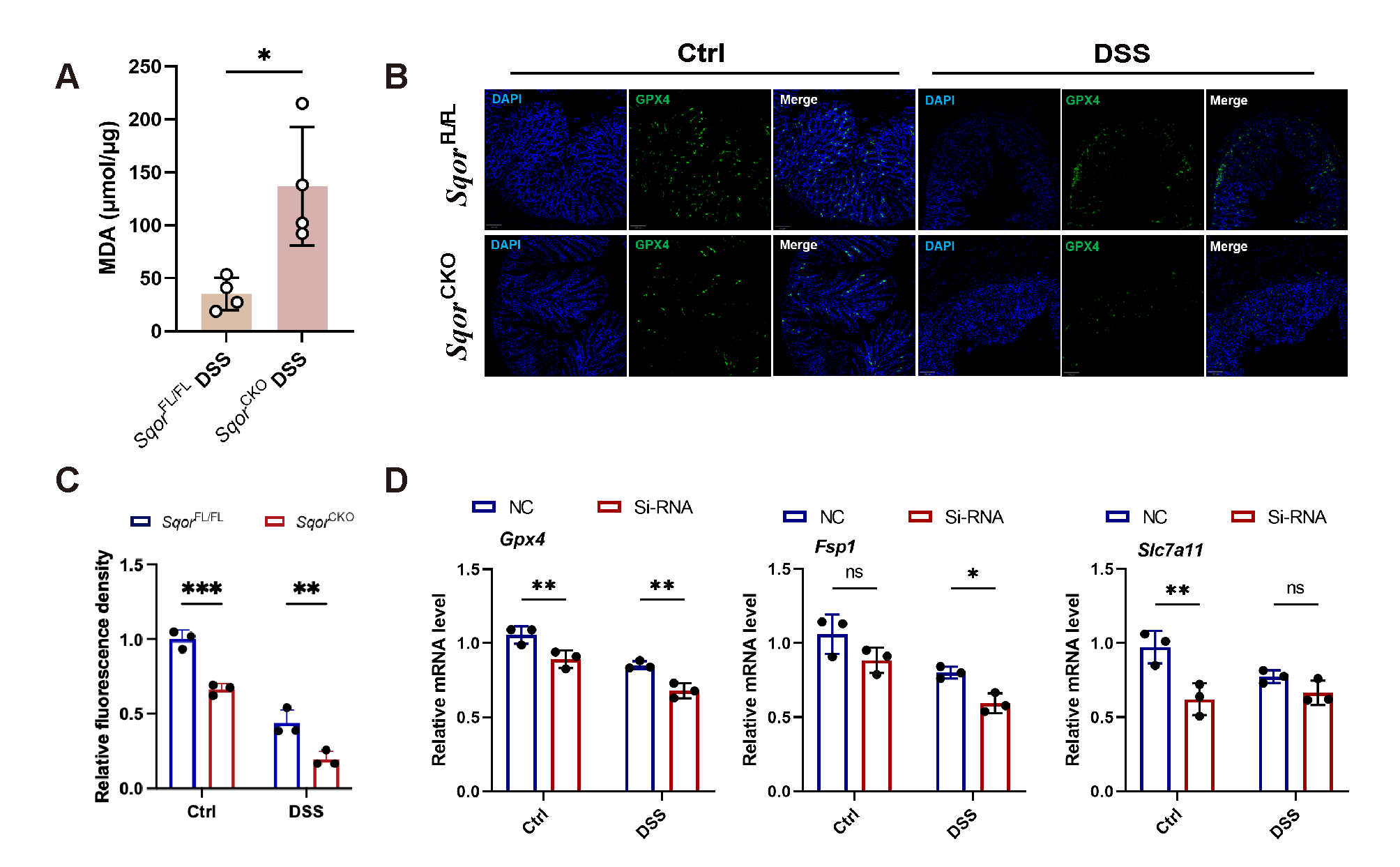
**

**Figure S8 SQOR deficiency aggravated ferroptosis in NCM 460 cells. (A)** Detection of MDA level in colonic tissues from *Sqor*^FL/FL^ and *Sqor*^CKO^ mice after DSS treated (n=4). (B-C) The GPX4 in the mouse colon sections were determined by immunofluorescence staining from *Sqor*^FL/FL^ and *Sqor*^CKO^ mice after DSS treated or not (scale bar: 100 μm). (D) The mRNA levels of Gpx4, Fsp1, and Slc7a11 in NCM460 cells with transfected with SQOR siRNA or negative control siRNA in the presence or absence of DSS. The data were represented as mean ±SD. n=3 per group. **P < 0.05, **P < 0.01, ***P < 0.001.* ns, no significant difference.

**
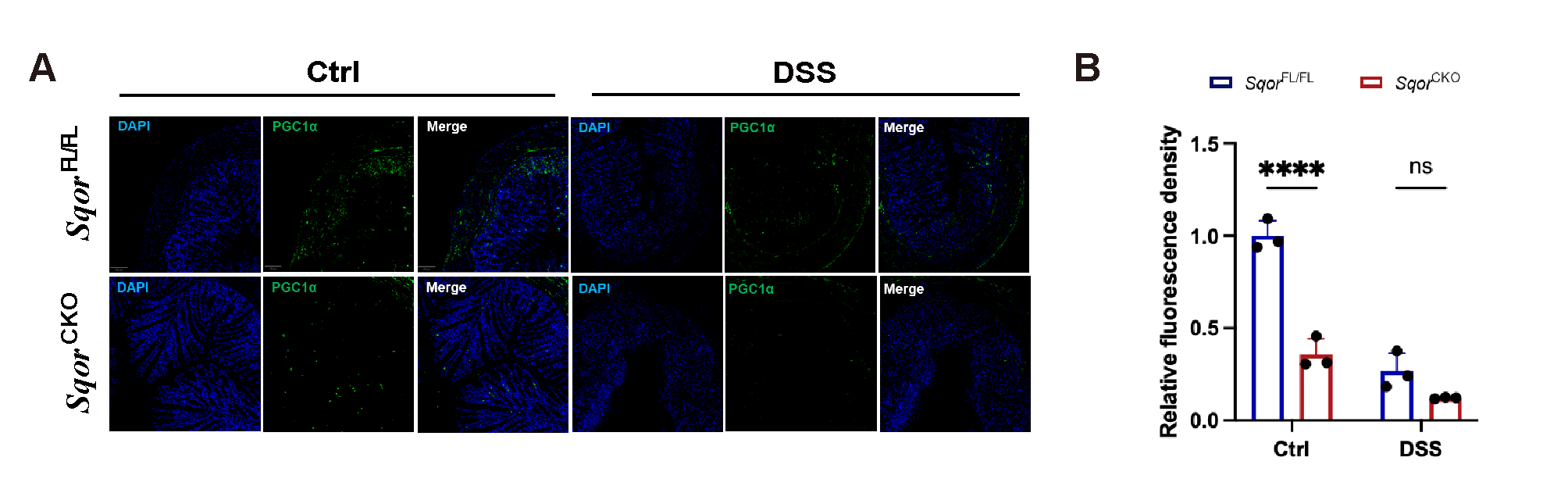
**

**Figure S9 Intestinal epithelial cells function is associate with ROS.** (A-B) The PGC1α in the mouse colon sections were determined by immunofluorescence staining from *Sqor*^FL/FL^ and *Sqor*^CKO^ mice after DSS treated or not (scale bar: 100 μm). The data were represented as mean ±SD. n=3 per group. *****P < 0.0001.* ns, no significant difference.

**Table S1. Primers applied for genotyping**

| **Primer** | **Sequence（5’-3’）** |
| --- | --- |
| SQOR-P1 | AGGGCAGAGGTCAGTTCCAAGTGC |
| SQOR-P2 | AGGCTATCTAGGACTATTTAAAGA |
| Villin-Cre -P1 | TTCTCCTCTAGGCTCGTCCA |
| Villin-Cre -P2 | CATGTCCATCAGGTTCTTGC |
| Villin-Cre -P3 | CTAGGCCACAGAATTGAAAGATCT |
| Villin-Cre -P4 | GTAGGTGGAAATTCTAGCATCATCC |

**Table S2. Primers applied for qRT-PCR**

| **Primer** | **Sequence（5’-3’）** |
| --- | --- |
| Mouse-TNFα-F | CCCACGTCGTAGCAAACCAC |
| Mouse-TNFα-R | GCAGCCTTGTCCCTTGAAGA |
| Mouse-IL-6-F | ACAAAGCCAGAGTCCTTCAGA |
| Mouse-IL-6-R | TGGTCCTTAGCCACTCCTTC |
| Mouse-IL-1β-F | TGACGGACCCCAAAAGATGA |
| Mouse-IL-1β-R | AAAGACACAGGTAGCTGCCA |
| Mouse-S100a9-F | ACCACCATCATCGACACCTTC |
| Mouse-S100a9-R | AAAGGTTGCCAACTGTGCTTC |
| Mouse-Cxcl1-F | GACCATGGCTGGGATTCACC |
| Mouse-Cxcl1-R | CGCGACCATTCTTGAGTGTG |
| Mouse-Ccl2-F | ACTCAAGCCAGCTCTCTCTT |
| Mouse-Ccl2-R | TTCCTTCTTGGGGTCAGCAC |
| Mouse-β-Actin-F | CCACCATGTACCCAGGCATT |
| Mouse-β-Actin-R | AGGGTGTAAAACGCAGCTCA |
| Mouse-Atp5a1-F | CATTGGTGATGGTATTGCGC |
| Mouse-Atp5a1-R | TCCCAAACACGACAACTCC |
| Mouse-Cox4i1-F | AGTGTTGTGAAGAGTGAAGAC |
| Mouse-Cox4i1-R | GCGGTACAACTGAACT |
| Mouse-Uqcrc1-F | ATCAAGGCACTGTCCAAGG |
| Mouse-Uqcrc1-R | TCATTTTCCTGCATCTCCCG |
| Mouse-Ndufab1-F | GGACCGAGTTCTGTATGTCTTG |
| Mouse-Ndufab1-R | AAACCCAAATTCGTCTTCCATG |
| Human-TNFα-F | ACCCTCACACTCAGATCATCTTC |
| Human-TNFα-R | TGGTGGTTTGCTACGACGT |
| Human-IL-6-F | GTAGCCGCCCCACACAGA |
| Human-IL-6-R | CATGTCTCCTTTCTCAGGGCTG |
| Human-IL-1β-F | TCAGGCAGGCAGTATCACTCA |
| Human-IL-1β-R | GGAAGGTCCACGGGAAAGAC |
| Mouse-COX2-F | ATAACCGAGTCGTTCTGCCAAT |
| Mouse-COX2-R | TTTCAGAGCATTGGCCATAGAA |
| Mouse-Rsp18-F | TGTGTTAGGGGACTGGTGGACA |
| Mouse-Rsp18-R | CATCACCCACTTACCCCCAAAA |
| Mouse-PGC1α-F | TATACTTTACGCAGGTCGAA |
| Mouse-PGC1α-R | ACAGAGAGTGTAAAGTAGGAG |
| Mouse-Nrf1-F | AGGAACCCTCAGTCTCACGA |
| Mouse-Nrf1-R | GTCCTCACAGAGTCTTCTGACAC |
| Mouse-Tfam-F | AGCTGGTGTTAGCATACGGA |
| Mouse-Tfam-R | GGTGGCAAATTCGGAAGAGG |
| Mouse-Gpx-F | GTCTCTCTGAGGCACGATCCG |
| Mouse-Gpx-R | TTCCGCAGGAAGGTAAACAGC |
| Mouse-Trx2-F | TGGGCTTCCCTCACCTCTAAG |
| Mouse-Trx2-R | CCTGGACGTTAAAGGTCGTCA |
| Mouse-Sod2-F | CAGACCTGCCTTACGACTATGG |
| Mouse-Sod2-R | CTCGGTGGCGTTGAGATTGTT |
| Mouse-Ucp2-F | ACTGTGCCCTTACCATGCTCC |
| Mouse-Ucp2-R | ATTGGTAGGCAGCCATTAGGG |
| Mouse-Ucp4-F | GAATGCCTATCGCCGAGGA |
| Mouse-Ucp4-R | AGTAGGAACTTGCTCGTCCGG |
| Mouse-Ucp5-F | TCCCAACTGCTCAGCGTG |
| Mouse-Ucp5-R | GGTGCTTCTTGGTAATATCATAAACG |
